# BioPAX in 2024: Where we are and where we are heading

**DOI:** 10.1016/j.csbj.2024.10.045

**Published:** 2024-11-04

**Authors:** Cécile Beust, Emmanuelle Becker, Nathalie Théret, Olivier Dameron

**Affiliations:** a Univ Rennes, Inria, CNRS, IRISA - UMR 6074, Rennes, F-35000, France; b Univ Rennes, Inserm, EHESP, Irset, UMR S1085, Rennes, F-35000, France

**Keywords:** Systems Biology, BioPAX, Biological pathways, Databases

## Abstract

In systems biology, the study of biological pathways plays a central role in understanding the complexity of biological systems. The massification of pathway data made available by numerous online databases in recent years has given rise to an important need for standardization of this data. The BioPAX format (Biological Pathway Exchange) emerged in 2010 as a solution for standardizing and exchanging pathway data across databases. BioPAX is a Semantic Web format associated to an ontology. It is highly expressive, allowing to finely describe biological pathways at the molecular and cellular levels, but the associated intrinsic complexity may be an obstacle to its widespread adoption.

Here, we report on the use of the BioPAX format in 2024. We compare how the different pathway databases use BioPAX to standardize their data and point out possible avenues for improvement to make full use of its potential. We also report on the various tools and software that have been developed to work with BioPAX data. Finally, we present a new concept of abstraction on BioPAX graphs that would allow to specifically target areas in a BioPAX graph needed for a specific analysis, thus differentiating the format suited for representation and the abstraction suited for contextual analysis.

## Highlights

• BioPAX meets its initial objectives.
• Pathway databases differ in scope and in BioPAX pathway representation.
• BioPAX' complexity hinders its adoption by biologists and reasoning tool developers.

## Graphical abstract

**Figure:**
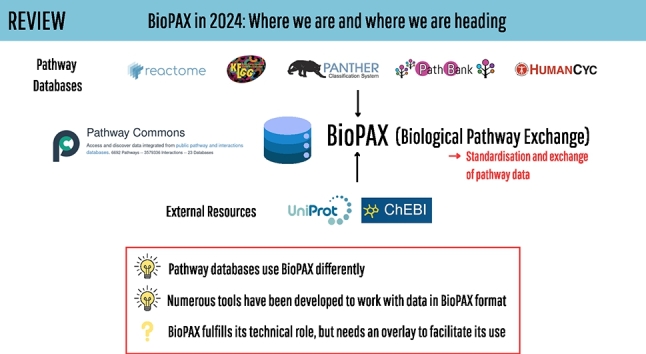

## 1 Introduction

Biological information encompassing data and knowledge is nowadays accumulating thanks to refinement of experimental methods in biology. This opens great possibilities for gaining new understandings of biological phenomenons. This information needs to be stored and shared online through databases, that could either represent general biological knowledge, like the Reactome pathway database (Jassal et al. [1], Milacic et al. [2]) or specific to some biological entities, like UniProtKB (Pundir et al. [3], The UniProt Consortium [4]) for proteins or ChEBI (Hastings et al. [5]) for small molecules. The large number of biological databases resulted in fragmented biological data across sources, and their schema heterogeneity hampers their joint analyses. The need of standards to represent biological knowledge thus emerged with data massification.

The COMBINE initiative (COmputational Modeling in BIology NEtwork) (Hucka et al. [6]) started in 2015 to harmonize efforts in systems biology. COMBINE is a common project proposing and coordinating several pre-existing standards to represent and model biological data according to specific goals. Modeling is covered by SBML (Systems Biology Markup Language) (Keating et al. [7], Lahti [8]). Graphical representation is covered by SBGN (Systems Biology Graphical Notation) (Le Novère et al. [9]). Exchange and interoperability is covered by BioPAX (Biological Pathway Exchange) (Demir et al. [10]), a standard language designed for the representation of biological pathways at the molecular and cellular levels. Other specific standards exist in the COMBINE initiative such as CellML (Cell Markup Language), (Cuellar et al. [11], Miller et al. [12]) for mathematical models or SBOL (Synthetic Biology Open Language) (Galdzicki et al. [13], Baig et al. [14]) for synthetic biology.

Pathways are a key notion in systems biology. A pathway is defined as a series of interactions between molecules that can be physical or genetic cellular components, describing a cause-and-effect or a time-dependant process, explaining an observable biological phenomenon (Demir et al. [10]). There exist several biological pathway databases but they do not share a common data format. This results in technical challenges concerning the standardization and integration of pathway data from heterogeneous sources and the development of methods and tools to help scientist access, map and analyze pathway information.

The BioPAX format aims to address several needs related to these challenges by making pathway data easier to collect, interpret and share. It allows the representation of pathway data using a controlled vocabulary to describe the pathways, their biochemical reactions, the molecules involved as well as regulation processes. It is designed to enable the integration of heterogeneous pathway data coming from multiple sources and their efficient sharing and interoperability. Moreover, BioPAX facilitates the storage and automatic querying of pathway data within databases, acting as a common interface for software tools to access pathway data from multiple sources (Demir et al. [15]). BioPAX emerged in 2002 with a first level supporting only metabolic pathway interactions. In 2005, the level 2 added support for molecular interactions and post-translational protein modifications. Since 2010, the level 3 also supports signaling pathways, molecular state, gene regulation and genetic interactions.

Here we report on the use of BioPAX across pathway databases and list some potential technical, organizational and conceptual issues that may limit its adoption by the bioinformatician or biologist communities, and suggest some areas of improvement to facilitate and extend the use of the BioPAX format. We first give an illustrated overview of the BioPAX format and examples of pathway represented in BioPAX. Then we compare how BioPAX is used across the main pathway databases and assess how these databases use BioPAX to cross-link and make their data interoperable. We also present the various tools that have been developed to work with BioPAX data. Finally, we propose an abstraction strategy to differentiate the data representation formalism and the data analysis formalism using BioPAX, paving the way to future possible applications of BioPAX data through the use of graph abstractions.

## 2 An illustrated overview of the BioPAX pathway data language

BioPAX is a standard language designed for the representation of metabolic, signaling pathways, molecular and genetic interaction as well as gene regulation networks (Demir et al. [10]). It focuses on exchanging and integrating large biological process maps (Hucka et al. [6]). BioPAX was developed and is maintained by a multi-disciplinary scientific community including pathway database groups (data providers and users) and tool developers. It evolves in the form of levels, each level refining the format or enriching it with new functionalities. The current level of BioPAX is the level 3 created in 2010. The BioPAX material and documentation is open source and accessible at http://www.biopax.org/.

Technically, BioPAX is a data format based on RDF (Resource Description Framework) and OWL (Ontology Web Language), Semantic Web formats (Bizer et al. [16]) supporting data interoperability and allowing to seamlessly take knowledge into account during processing. Entities and relationships from pathway data described in BioPAX are represented in the form of RDF triples with a subject (from which the relation starts), a relation (a predicate) and an object (to which the relation points to). BioPAX data can thus be represented as directed graphs, that can be automatically queried using the SPARQL language (Cheung et al. [17]).

BioPAX comes with an ontology providing a controlled vocabulary and a hierarchical system of classes and properties. Entities are instances of some BioPAX ontology classes, and the relations between entities are represented by BioPAX ontology properties.

**Fig. 1:**
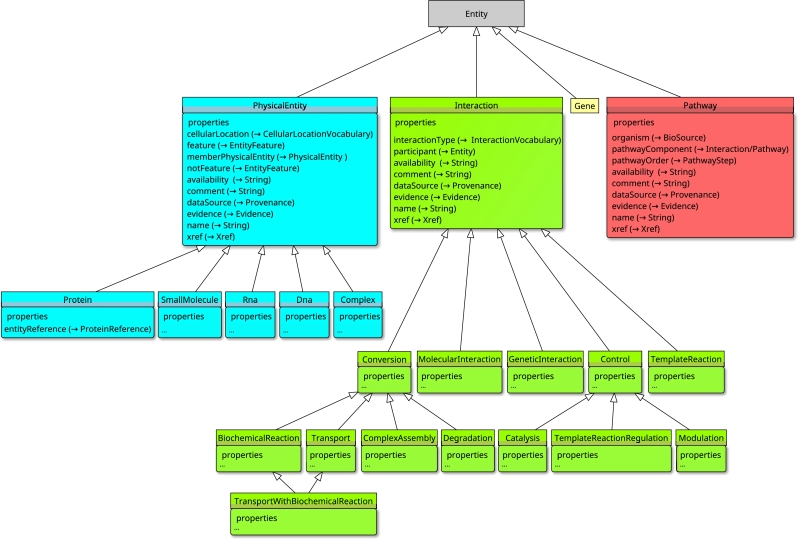
**Simplified BioPAX data schema adapted from Demir et al.****[10]****.** The hierarchy of the main classes of the BioPAX ontology is represented as color boxes. The main properties associated to each class are detailed at the bottom of the boxes. The types of resources properties point to are indicated next to each of them. The properties originating from a class are either class-specific or inherited from the ancestor classes. The Entity class (in gray) is the root of the BioPAX ontology. PhysicalEntity (in blue) describes the biological entities involved in pathways such as proteins, complexes, small chemical molecules or even RNA or DNA. Interaction (in green) encompasses several types of biological interactions including reactions as well as regulation processes that typically involve one or several physical entities. Pathway (in red) is used to characterize biological pathways, including the reactions and pathway steps (the sequence of reactions) composing them.

Fig. 1 represents a simplified diagram of the BioPAX ontology. It presents the main classes of the BioPAX ontology as well as the relationships (properties) that the instances of the classes can share to express their interactions. The Pathway class is used to describe among others the components of the pathways and their sequence of steps. The Interaction class allows to specify the nature of interactions that the entities share and allows to describe them according to their types (for example biochemical reactions, catalysis, molecular interactions...). The PhysicalEntity class is used to describe entities, including the specification of their nature (proteins, small molecules, complexes, RNA or DNA). Other classes exist in the BioPAX ontology but are not represented on Fig. 1, like the Xref class which allows to link external references to the entities (Pubmed identifiers of publications, cross-links to other databases, identifiers...). The complete diagram of the BioPAX ontology is provided as Supplementary Figure S-1.

**Fig. 2:**
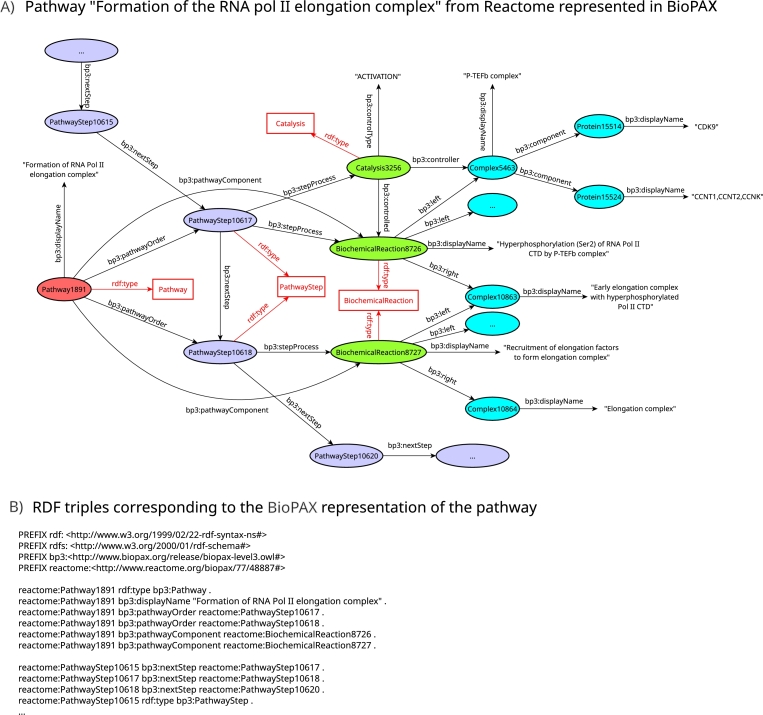
**Example of a pathway represented in BioPAX.** (Panel A) The pathway represented is the ‘Formation of the RNA Poll II elongation complex’ pathway (R-HSA-112382) from the Reactome database (version 90, September 2024). It is described using the classes and properties of the BioPAX ontology. Each arrow represents a relation (also called a predicate) from a subject to an object, the whole making up a RDF triple (Panel B, see Supplementary File S-2 for the complete list of RDF triples). Resources are represented with their identifiers in the BioPAX export of Reactome (version 87). Instances are represented by ellipses and classes by boxes (node color legend from Fig. 1). The instantiation relationships describing the membership of resources to a class are represented by red arrows. Instantiation relationships were omitted for the PhysicalEntity instances. The pathway of interest (in red) is composed of two direct pathway steps (in purple), linked by a bp3:nextStep relationship indicating the sequence of the steps. The first pathway step (PathwayStep10617) is composed of the BiochemicalReaction8726 (‘Hyperphosphorylation (Ser2) of RNA Pol II CTD by P-TEFb complex’) which consumes the Complex5463 (via the bp3:left relation) and produces the Complex10863 (via the bp3:right relation). BiochemicalReaction8726 is controlled (activated) by the Catalysis3256 reaction. The second pathway step (PathwayStep10618) is composed of the BiochemicalReaction8727 (‘Recruitment of elongation factors to form elongation complex’) which consumes the product of the previous pathway step and produces the Complex10864.

For a better understanding, an illustration of use of the classes and properties of the BioPAX ontology with a pathway example is given in Fig. 2 (panel A), where we present the instantiation of a pathway from Reactome (Jassal et al. [1]) (*‘Formation of RNA pol II elongation complex’*, R-HSA-112382). The pathway is represented as a directed graph describing the interactions of the pathway components, their types and their associated biochemical reactions. The nodes representing entities are drawn as ellipses and nodes representing BioPAX classes are drawn as boxes. Node colors match those of the ontology presented in Fig. 1. The ellipse corresponding to the pathway of interest is represented in red, it is an instance of the Pathway class. The bp3:pathwayOrder property is used to specify the composition of the pathway into pathway steps (in purple ellipses, instances of the PathwayStep class). The bp3:nextStep property specifies the sequence organization of the pathway steps. Each pathway step is associated to one or more interactions (green ellipses, instances of sub-classes of Interaction) by the bp3:stepProcess property. Typically, it allows to group a biochemical reaction and its associated catalysis as a pathway step. Here for the sake of representation, we only detailed the interactions linked to the PathwayStep10617 and PathwayStep10618. The other instances of PathwayStep are represented to show the interconnections of pathways in Reactome. Here in the PathwayStep10617, the Catalysis3256 interaction activates the BiochemicalReaction8726 via the bp3:controlled property. Each interaction in the graph (green ellipses) is linked to entities that can be the reactants (property bp3:left), products (property bp3:right) or the controllers (property bp3:controller) of a biochemical reaction. These entities can be any type of instance of the PhysicalEntity class of the BioPAX ontology. Here some complexes being part of the reactions are detailed (with blue ellipses). The proteins composing these complexes are specified with the bp3:component property. All the entities and relationships visualized on the BioPAX graph are encoded as RDF triples such as illustrated on the panel B of Fig. 2. The complete list of RDF triples corresponding to the pathway is given in Supplementary Figure S-2. The pathway was extracted from the BioPAX export of Reactome (version 90, September 2024), using a SPARQL query provided as Supplementary Figure S-3.

The BioPAX ontology thus provides a controlled vocabulary, and allows to finely describe, in a standard way, the mechanisms connecting the multiple elements involved in biological processes. BioPAX data can be queried using the SPARQL query language, in order to retrieve specific triples, specific entities (nodes) or specific relationships (edges) from the graph. The explicit and fine-grain representation of information supports highly-expressive queries and powerful analyses, but can quickly become complex as the pathways grow in size.

## 3 BioPAX as a common but flexible pathway language

The goal of the BioPAX format is to describe biological pathway data in an uniform way. To do so, BioPAX comes with a controlled vocabulary and semantics, establishing a standard way to represent pathway information. However, this standardization effort can only be fully effective if its flexibility is limited enough so that the vocabulary is used uniformly by the different providers.

To evaluate the uniformity in the production of BioPAX, we compared the BioPAX exports of five main pathway databases: Reactome (Jassal et al. [1]), PANTHER Pathway (Mi and Thomas [18], Thomas et al. [19]), PathBank (Wishart et al. [20], Wishart et al. [21]), HumanCyc (Romero et al. [22]) and KEGG Pathway (Kanehisa and Goto [23], Kanehisa et al. [24]). Except for the standalone BioPAX export of Reactome (version 90, September 2024) and the standalone export of PANTHER Pathway, all the BioPAX files of pathway databases were downloaded from the PathwayCommons aggregation service (PathwayCommons version 14, 2024), (Rodchenkov et al. [25]). PathwayCommons is part of the standardization effort of pathway data and aims to provide a platform for sharing pathway data files in a standardized format including BioPAX, PSI-MI (Kerrien and Hermjakob [26]) and SBML (Hucka et al. [27]). Because human is the only organism supported by all resources, our study compared human BioPAX exports. For the standalone export of PANTHER Pathway, which integrates information about protein orthologs, we filtered it in order to only retrieve information about human proteins in pathways.

Counts were done using SPARQL queries, that are available in a git repository (https://github.com/CecileBeust/BioPAXReview2024Codes). A data acquisition procedure description is available as Supplementary Text S-1.

For each pathway database, we represented the proportion of instances of eight classes from the BioPAX ontology on radar plots. We normalized the counts of instances by the maximal values of each class. This comparison highlights a high heterogeneity in the BioPAX exports of pathway information across databases, both in terms of content or representation. The scope of PANTHER Pathway (standalone BioPAX export and PathwayCommons export), HumanCyc, and KEGG Pathway seem to focus more on small molecules than on other instances types. PathBank has a more balanced focus between small molecules, interactions, pathways, pathway steps and biochemical reactions. Overall, Reactome presents more instances of biochemical reactions and physical entities (Rna, Dna and proteins) than every other databases. These differences in usage of BioPAX classes between pathway databases can be explained by the differences of scope between the databases. Indeed, the HumanCyc and KEGG Pathway are more focused on metabolism, that could explain why we observe more instances of SmallMolecule. Strikingly Reactome and PathBank are the only resources using the PathwayStep class to describe a unit composing a pathway that can belong to several pathways, interconnecting pathway graphs. Other pathway databases make little or no use of the PathwayStep class, but represent a pathway as an independent unit of information, in which the components are not described as sequential reactions. This observation suggests that pathway databases are more or less detailed, leading to a different low-level representation. Reactome is thus the most comprehensive and connected map of biological processes.

**Fig. 3:**
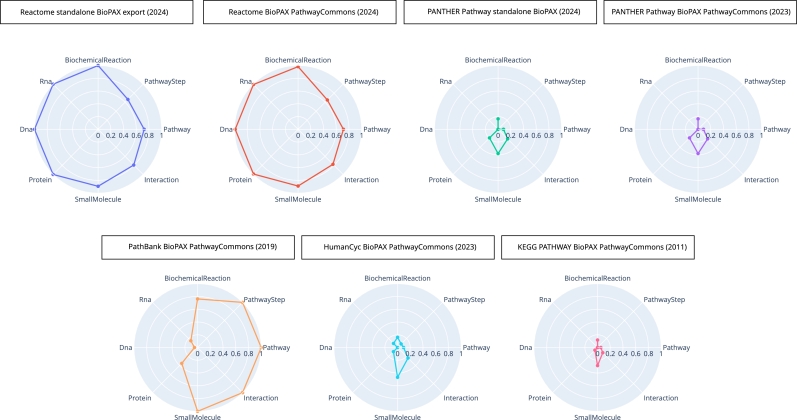
**Comparison of the contents of BioPAX exports of the main biological pathway databases.** The BioPAX exports of five pathway databases were extracted from the PathwayCommons database (Reactome, PANTHER Pathway, PathBank, HumanCyc, and KEGG Pathway) and the standalone BioPAX export of Reactome (version 90, September 2024) and PANTHER Pathway have been added. For each database, the number of instances of each of the following BioPAX class is represented on the radar plots: BiochemicalReaction, PathwayStep, Pathway, Interaction, SmallMolecule, Protein, Dna, Rna. The values are calculated as percentages of the maximum value of each class. The same comparison was extended to the nine pathway databases available on PathwayCommons is available in Supplementary Figure S-4. For more specific information on each pathway database, a detailed table is available as Supplementary Table S-1.

However, these results should not be generalized to the original resources before their export to BioPAX. First of all, the BioPAX files from the PathwayCommons database used in this study are not all up-to-date with the current versions of the databases. Only Reactome and PANTHER Pathway provide regular BioPAX exports independently from the PathwayCommons database (Reactome provides updated BioPAX exports every three months). The conversion tools used to convert databases to BioPAX are not mentioned on the PathwayCommons website. Another important point is that we can not distinguish between information that are not present in the original resources before conversion to BioPAX and information that is lost during conversion. We can distinguish three different types of information that can be lost during the conversion to BioPAX: the information that could have been translated to BioPAX but was not present in the original database, the information that could have been translated to BioPAX but was not because of the converter, and the information that could not have been translated to BioPAX because of a specific type of information not supported by BioPAX. Keeping in mind that the differences in BioPAX classes' usage observed in Fig. 3 can be due to the differences in scope from the databases, these BioPAX exports are the only ones available for these different databases and their comparison remains relevant.

## 4 BioPAX as a language for pathway data exchange and interoperability

One of the goals of BioPAX is to make pathway data both reusable and extendable by external resources. Several features of BioPAX based on its RDF syntax address these requirements.

**Fig. 4:**
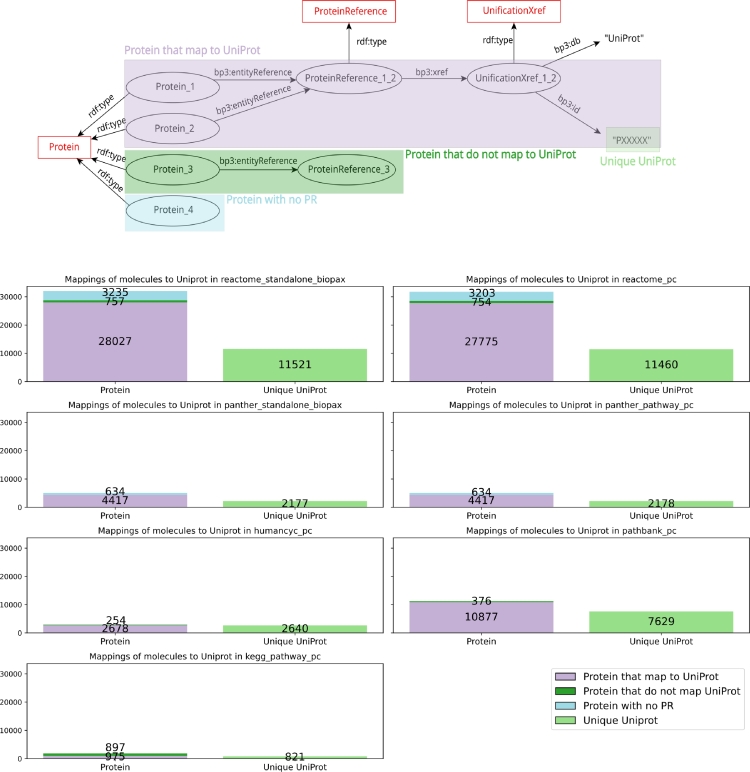
**Mappings of BioPAX instances to UniProtKB in the BioPAX exports of the main pathway databases** (Reactome standalone BioPAX export and from PathwayCommons, PANTHER Pathway standalone BioPAX export and from PathwayCommons (filtered for human-only uniprot protein mappings), HumanCyc from PathwayCommons, PathBank from PathwayCommons and KEGG Pathway from PathwayCommons). The top panel represents the mapping of BioPAX proteins to UniProtKB. Each instance of Protein (P) points to a ProteinReference (PR) that can be linked to a UniProtKB identifier. On the bottom panel, the number of mappings from the BioPAX instances of P to UniProtKB is detailed as well as the number of the unique UniProtKB identifiers that the database points to. The number of instances of P that lack association with PR is also reported, as well as the number of P whose PR do not point to a UniProtKB identifier. Comparative analysis for the nine pathway databases available on PathwayCommons is provided as Supplementary Figure S-6.

**Fig. 5:**
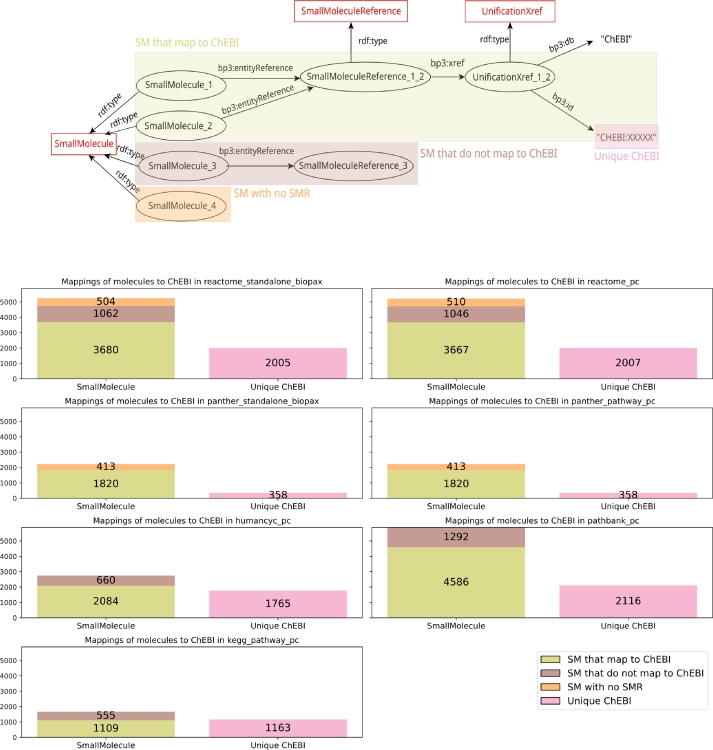
**Mappings of BioPAX instances to ChEBI in the BioPAX exports of the main pathway databases** (Reactome standalone BioPAX export and from PathwayCommons, PANTHER Pathway standalone BioPAX export and from PathwayCommons, HumanCyc from PathwayCommons, PathBank from PathwayCommons and KEGG Pathway from PathwayCommons). The top panel represents the mapping of BioPAX small molecules to ChEBI. Each instance of SmallMolecule (SM) points to SmallMoleculeReference (SMR) that can be linked to a ChEBI identifier. On the bottom panel, the number of mappings from the BioPAX instances of SM to ChEBI is detailed as well as the number of the unique ChEBI identifiers that the database points to. The number of instances of SM that lack association with SMR is also reported, as well as the number of SM whose SMR do not point to a ChEBI identifier. Comparative analysis for the nine pathway databases available on PathwayCommons is provided as Supplementary Figure S-7.

BioPAX graphs can be cross-linked with other databases thanks to the universal nature of RDF identifiers. Any dataset that refers to a BioPAX pathway or one of its components simply has to reuse its identifier, for example to represent the fact that it is involved in a particular disease. Conversely, a BioPAX dataset can refer to third-party resources to provide additional information. Typically, a BioPAX dataset would describe the participation of certain proteins and molecules to a particular pathway, and reference their identifiers in external authoritative resources such as UniProtKB (Pundir et al. [3], The UniProt Consortium [4]) or ChEBI (Chemical Entities of Biological Interest, Hastings et al. [5]), that respectively provide additional descriptions of proteins and small molecules. Fig. 4, Fig. 5 present the comparison of the entities mappings to UniProtKB and ChEBI respectively. To our knowledge, such comparison of pathway databases mappings has not already been performed.

Examples of BioPAX mappings of a protein to UniProtKB and a small molecule to ChEBI are respectively given in the top panels of Fig. 4, Fig. 5. One key element of these mappings is the use of the EntityReference class, which is used to group several entities across different contexts and molecular states. For example, a protein can exist in multiple molecular states (localization, post-translational modification, oligomerization...). Each of them has its own identifier in the BioPAX dataset and they appear as many instances of the Protein class. However, they are all associated to the same entity reference with the bp3:entityReference property to represent the fact that they are different manifestations of the same protein. The same mechanism exists for small molecules.

BioPAX then allows to provide cross links from entity references to external databases such as UniProtKB, ChEBI or PubMed via the bp3:xref property. A mapping of an entity from the BioPAX graph and an external resource is thus specified by a path in the graph that starts from the entity (protein or small molecule), goes through its corresponding entity reference and points to an instance of UnificationXref related to a UniProtKB or a ChEBI identifier. The mapping process to UniProtKB proteins for the PANTHER Pathway database is slightly different. Because the PANTHER Pathway database integrates information about protein families and orthologs, the database sets the UniProtKB mappings by passing through the bp3:memberEntityReference properties. A schema explaining the mapping process to UniProtKB in PANTHER Pathway is available as Supplementary Figure S-5.

As shown in the bottom panels of Fig. 4, Fig. 5, we counted the number of proteins that map to a UniProtKB identifier and the number of small molecules that map to a ChEBI identifier for the six different BioPAX exports of pathway databases. When these mappings were correctly set up, we counted the number of unique UniProtKB and ChEBI identifiers. Conversely, when the mappings are not set up, we enumerated the number of entities that do not map to UniProtKB or ChEBI, either because they are not associated to an instance of entityReference or because their reference do not map at all to these external resources.

For the mappings to UniProtKB, the number of proteins that are cross-linked to UniProtKB is the highest (28027) for the standalone BioPAX export of Reactome (version 90, September 2024) and is of 27775 for the PathwayCommons export of Reactome. The number of mappings is lower for the other databases (lower than 11000). This can partly be explained by the huge difference between the number of protein entities in Reactome and the other databases. Taken as percentages, the proportion of proteins that map to UniProtKB is higher than 85% for the two versions of Reactome, for HumanCyc and for PathBank (highest score with 96.6% of mappings). This demonstrates an overall good mapping of BioPAX entities to UniProtKB.

For the mappings to ChEBI, we observe that the number of small molecules cross-linked to ChEBI is the highest for PathBank (4586), followed by Reactome (with 3680 mappings in the standalone BioPAX export and 3667 mappings in the PathwayCommons export). The number of mappings is lower for the other databases (lower than 4000). This may be explained by the higher number of small molecules present in the PathBank database and also in Reactome. Taken as percentages, the proportion of small molecules that map to ChEBI is higher than 75% for the two PANTHER Pathway exports, for PathBank and HumanCyc, and is close to 70% for the other databases.

Overall, the proportion of entities that map UniProtKB or ChEBI is greater than the proportion of entities that do not map, highlighting an interconnection of BioPAX pathway data with existing other resources. We noticed that for every database, the number of unique identifiers is lower than the number of mappings. It highlights the granularity of the representation of entities in BioPAX: entities are described individually in different molecular states, but are also gathered with references, which are used to set the mappings. Interestingly, we remark that only the latest BioPAX export of Reactome presents entities that are not associated to an instance of entityReference. These proteins that are not associated to a reference in Reactome are converted from EntitySets. Reactome uses EntitySets to gather physical entities that function interchangeably in a given situation. For example, the EntitySet “Insulin-like growth factors” is not found in UniProtKB, but gathers two proteins (IGF-1 and IGF2(25-91)), which map to UniProtKB in the BioPAX export.

Overall, for Reactome, PANTHER Pathway, HumanCyc and PathBank, entities described in BioPAX are well mapped to UniProtKB and ChEBI (mappings greater than 70%). This mapping is supported by the use of the entityReference and Xref classes of the BioPAX ontology. Cross-links between BioPAX entities and UniProtKB and ChEBI strengthen interoperability of resources as these bases act as references for proteins and small molecules.

However, some potential improvements in the mapping process could increase interoperability. The presence of entities that do not map to UniProtKB and ChEBI can be explained by a lack of harmonization in the use of identifiers across databases, which can lead to incomplete mappings and reduced interoperability. Another lack of uniformity in these mappings is that the character strings referring to UniProtKB and ChEBI in the BioPAX files are not the same from one database to the other. The standalone export of Reactome (version 90, September 2024) uses the strings “UniProt” and “ChEBI” where other databases use the strings “uniprot” and “chebi”. This is an obstacle to the standardization of query protocols when we want to extract knowledge from BioPAX files. Using the same identifiers in all databases would both improve integration of BioPAX data coming from different sources and reduce redundancy.

## 5 Using BioPAX: a landscape of available tools

Since the creation of BioPAX, a wide range of tools and packages have been developed to work with BioPAX files. Due to the intrinsic complexity of the BioPAX format, specialized tools are needed to browse, retrieve, visualize and analyze pathway data in BioPAX. An overview of the landscape of available tools allowing to work with BioPAX files is available as a mindmap in Supplementary figure S-8. Also, a table of these tools is provided as Supplementary Table S-2.

One of the most polyvalent tool to work with BioPAX files is Paxtools (Demir et al. [15]). Paxtools is an open-source Java library that constitutes the only complete BioPAX API available. It allows to perform common but complicated tasks on BioPAX files such as reading, writing, searching, merging, comparing and transforming pathway data contained in BioPAX files.

Although Paxtools is a quite complete and efficient tool for manipulating BioPAX files, it remains difficult to use for non computer scientists or users unfamiliar with Java programming or command-line based tools. Some tools have been developed in order to simplify the access to specific pathway information, or allow the manipulation of BioPAX files with other common languages in bioinformatics.

A Python tool allowing to work with BioPAX files is PAX2GRAPHML (Moreews et al. [28]). It allows to interpret BioPAX files as regulated reaction graphs describing biochemical reactions with their substrates, products, and regulators. PAX2GRAPHML allows to analyze pathway data and extract sub-graphs. The PyBioPAX Python package (Gyori and Hoyt [29]) was recently created in order to meet the need of a Python software support for BioPAX. PyBioPAX allows to manipulate and analyze BioPAX models by implementing the BioPAX model as a set of Python classes. Finally, easy manipulation of BioPAX files will be improved with the coming release of the Python BIOPAX-Explorer package (https://forgemia.inra.fr/pegase/biopax-explorer), which will provide a BioPAX Python object-model with advanced query features.

Working with BioPAX files in R is possible with the R package rBiopaxParser (Kramer et al. [30]), which allows to build regulatory networks from pathway information. Also, a part of the Paxtools library has been exported to an R package named PaxtoolsR (Luna et al. [31]).

Some tool aim to facilitate the access of pathway information in BioPAX, like the BioPAX-Parser BiP (Agapito et al. [32]) which is available through a graphic command and aims to allow users easily explore and analyze pathway information. It allows to perform pathway enrichment analysis (PEA).

Some other tools aim to meet the need of providing a visual representation of BioPAX models for easier pathway visualization. ChiBE (Chisio BioPAX Editor) (Babur et al. [33]) is a software that allows easy manipulation and visualization of BioPAX models. The Cytoscape application (Shannon et al. [34]) is able to read and visualize BioPAX files. Although Cytoscape plugins were developed to reason on BioPAX data using all the properties of the language (like the BiNoM plugin (Bonnet et al. [35])), they are unfortunately not maintained. NAViGaTOR (Network Analysis, Visualization, & Graphing TORonto) (Brown et al. [36]) is also a software for visualizing and analyzing networks that supports BioPAX files. CellDesigner (Funahashi et al. [37]) is a software that allows to create pathway diagrams. It comes with a package that is able to export pathway data in BioPAX Level 3 (Mi et al. [38]). The PathVisio software (Kutmon et al. [39]) also has a BioPAX plugin, allowing to import and export pathway data in BioPAX.

BioPAX files can be converted to other data formats using specific tools, thus improving its interoperability with the other formats and softwares that use these formats. As there are no general tools allowing to directly reason on BioPAX data, a conversion step to another data format is often needed for algorithms or tools to be used. Paxtools (Demir et al. [15]) and ChiBE (Babur et al. [33]) allow to convert BioPAX files to other data formats, ensuring wider potential downstream analysis. BioPAX data can be converted to SBML (Systems Biology Markup Language) (Keating et al. [7]) using for example the BIOPAX2SBML converter (Büchel et al. [40]), allowing integration with SBML models and dynamic simulation of the system. BioPAX data can also be converted into SBGN (Systems Biology Graphical Notation) (Le Novère et al. [9]), a graphical language allowing to build alternative projections of complex biological data.

BioPAX files can also be converted in other data formats that are not standards. For example it is possible to convert BioPAX data to GRAPHML files using PAX2GRAPHML (Moreews et al. [28]). It is also possible to convert BioPAX files in formats suitable for dynamical modeling. For example, Vignet et al. [41] developed a package, biopax2cadbiom, that automatically generates Cadbiom models from BioPAX files, allowing to perform dynamical modeling on large-scale networks. Considering the conversion of original pathway data to BioPAX, this process is often realized independently by resource providers. Most of the time, this conversion has to be set up manually and adapted to the type of data the database describes, as done by Lee et al. [42].

Finally, one singular tool available in the landscape of BioPAX tools is the BioPAX Validator (Rodchenkov et al. [43]). It allows to detect non-conformities in BioPAX files, by identifying issues in a BioPAX document. However, the BioPAX validator only validates the syntactic aspect of BioPAX. Currently there exists no tool capable of assessing the semantics of pathway data within BioPAX files. Inconsistencies are frequent in BioPAX files. For example, widespread invalid description of molecular complexes has been shown in the BioPAX export of Reactome (Juigné et al. [44]). Improving the semantic validation of BioPAX data is a key point to reduce the non-conformities in the usage of BioPAX across pathway databases.

Overall, despite the variety of tools developed for handling BioPAX data, the format remains poorly used by the scientific community. The lack of BioPAX user-friendly interfaces for pathway analysis is one of the reasons. Also, some of the tools presented above have a small user community, making them difficult to use. Another important point is that there are not enough tools allowing to directly reason on BioPAX data and extract knowledge from it. The majority of tools are more adapted for the querying of the files, like for example getting the RDF triples associated to a pathway of interest. These tools are not suited for direct reasoning on BioPAX data, such as traversing the graph of pathways or identifying potential regulators of biochemical reactions.

## 6 Interfacing BioPAX: from low-level representations to high-level abstractions

As illustrated in Fig. 3, the information in BioPAX exports can vary depending both on the scope of databases, and on the way the BioPAX semantics is used. There exist several representations of the same data in BioPAX, which can be seen as low-level representations of raw BioPAX data.

Based on the low-level representation of the pathway ‘Signaling by EGFR’ (R-HSA-177929) from Reactome, we show an abstraction method to shield users from the complexity of the BioPAX graph. Note that we envision this abstraction as a post-processing of BioPAX data in order to meet a specific goal, not as a replacement for BioPAX.

As shown in Fig. 6, the pathway is finely described using the classes and properties of the BioPAX ontology, resulting in a dense directed graph (323 nodes and 576 edges). The root pathway ‘Signaling by EGFR’ is composed of five subpathways linked to the root pathway with the bp3:pathwayComponent property. The same property is also used to link pathways to the biochemical reactions they are composed of. Each biochemical reaction is then associated to a reactant (bp3:left property) and a product (bp3:right property). The reactants and products are also described (small molecules, proteins or even complexes). Moreover, the regulation processes like catalysis associated to biochemical reactions are described (bp3:controller property), adding more relationships to the graph.

**Fig. 6:**
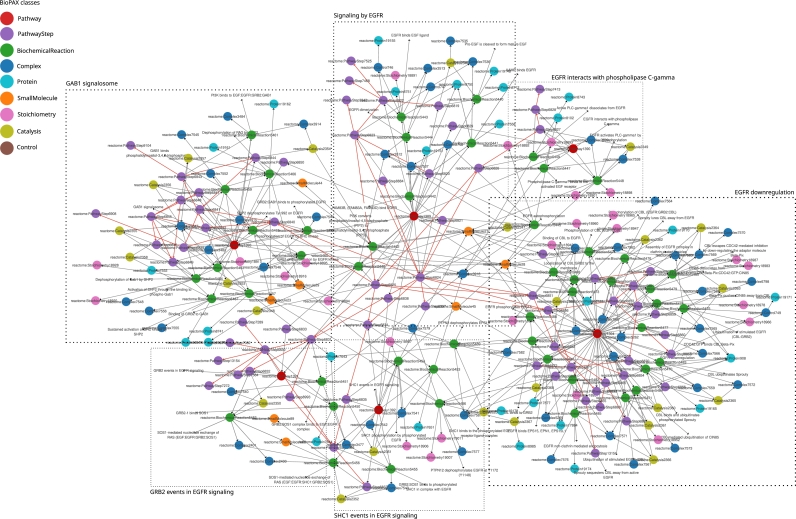
**Simplified BioPAX representation of the pathway ‘Signaling by EGFR’ (R-HSA-177929) from Reactome (BioPAX export (version 90, September 2024**)). The instances of some BioPAX classes are represented as color nodes (Pathway, PathwayStep, BiochemicalReaction, Complex, Protein, SmallMolecule, Stoichiometry, Catalysis, Control). The BioPAX properties between two resources are detailed on the edges. The root pathway ‘Signaling by EGFR’ and its five associated subpathways are framed with dashed boxes. Each of these pathways may be composed of different pathway steps (visualized as purple nodes), that are linked to processes that can be biochemical reactions (green nodes) or catalysis events that control the reactions (light green nodes). Red edges represent the bp3:nextStep edges between pathway steps. Each biochemical reaction involves entities as reactants or products, that can be complexes (dark blue nodes), proteins (light blue nodes) or small molecules (orange nodes). Biochemical reactions are also characterized by a stoichiometry, represented by pink nodes. The following BioPAX properties are not represented: bp3:dataSource, bp3:comment, bp3:xref, controlType, bp3:conversionDirection, bp3:evidence, bp3:organism, bp3:availability, bp3:entityReference, bp3:cellularLocation. Visualization using Cytoscape (Shannon et al. [34]).

For the sake of representation, the full pathway is not represented on Fig. 6. Some properties are omitted, such as the bp3:conversionDirection property of biochemical reactions which adds a hub node on the graph or the bp3:comment property which allows to link entities to curator's string comments. Also the cross-links to literature references and mappings external resources are not represented (bp3:xref property) as well as the provenance of entities (bp3:dataSource property) and their links to entity references (bp3:entityReference property). Thus, the representation of the ‘Signaling by EGFR’ pathway is already a simplification of the complex BioPAX representation of the pathway described in Reactome.

However, this low-level representation is still hard to visualize and analyze because of the density and quantity of information. For example, to retrieve the sequence of events that compose a pathway, it is necessary to pass through the PathwayStep instances and follow the bp3:nextStep properties. The use of the PathwayStep BioPAX class increases the number of paths to follow to access the sequence of biochemical reactions within pathways. Also, BioPAX introduces some bias in the topological analysis of the pathway graph. For example, every resource in a BioPAX graph is linked to an instance of the Provenance class via a bp3:dataSource property, indicating the source of the data (here the Reactome database). Each entity in the graph shares a minimal distance of two with other entities in the low-level BioPAX graph. This is a critical problem when computing shortest paths between entities, or applying random walk algorithms. There is a need to separate the BioPAX representation formalism (which we consider better than the alternatives) and the analysis formalism.

A solution can be to abstract the original low-level representation of the BioPAX data to generate high-level views of the original graph. For example, we can abstract the BioPAX graph of the ‘Signaling by EGFR’ pathway presented in Fig. 6 by abstracting the instances of the PathwayStep class. A first simplification of the BioPAX graph, only representing the instances of the Pathway, PathwayStep and BiochemicalReaction classes, is given in Supplementary Figure S-9. Still, this simplification of the graph does not allow to directly access to the sequence of biochemical reactions in the root pathway and its sub-pathways. A simplification is thus not enough to show this information, and an abstraction strategy based on reasoning is needed.

As shown in Fig. 7, the pathway ‘Signaling by EGFR’ from Fig. 6 has been abstracted to represent the sequence of biochemical reactions of the pathway (16 nodes and 21 edges). The direct biochemical reactions of the root pathway ‘Signaling by EGFR’ are represented (green nodes). Two biochemical reactions are linked by a plain green edge if their associated pathway steps on Fig. 6 (purple nodes) are linked by a bp3:nextStep property. This new relation on the abstracted graph has the URI abs:NextStepBiochemicalReaction. Biochemical reactions are linked to sub-pathways (red nodes) by a dashed gray edge if the following biochemical reaction of the sequence is a part of the sub-pathway. If any biochemical reaction of a sub-pathway is followed by a biochemical reaction of another sub-pathway, a dashed red edge is added between the sub-pathways. These two new relations have the URI abs:NextStepPathway. The abs prefixes stand for http://abstraction/.

**Fig. 7:**
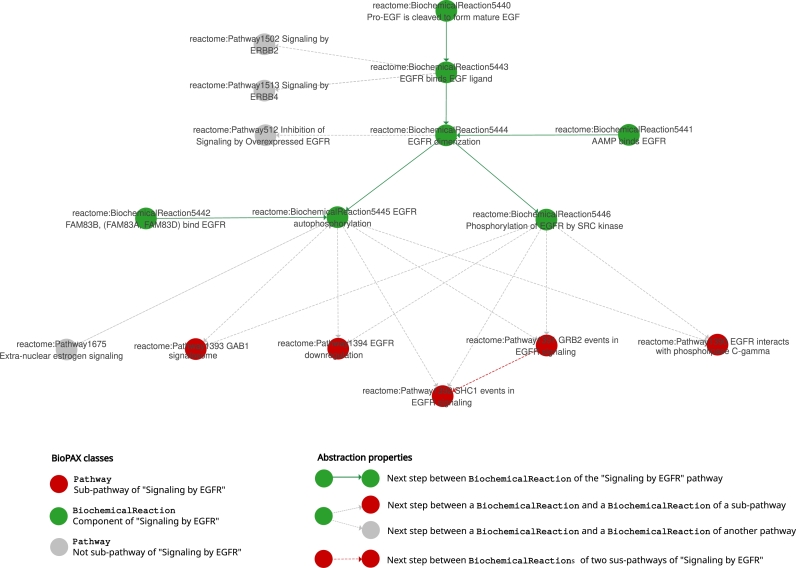
**Example of abstraction of the ‘Signaling by EGFR’ pathway (R-HSA-177929) from Reactome (version 90, September 2024**) of Fig. 6. The pathway was abstracted in order to show the sequence of biochemical reactions (green nodes) of the pathway and their interconnections to other sub-pathways (red nodes). On the original graph of Fig. 6, if two instances of PathwayStep are linked by a bp3:nextStep property, their associated biochemical reactions (green nodes) are linked by a new abs:NextStepBiochemicalReaction property (plain green edge) on the abstracted graph. If any biochemical reaction of the pathway is followed by a biochemical reaction belonging to another sub-pathway, an abs:NextStepPathway property (dashed gray edge) is added between the biochemical reaction node (green nodes) and the sub-pathway node (red nodes). If any biochemical reaction of a sub-pathway is followed by a biochemical reaction of another sub-pathway, an abs:NextStepPathway property (dashed red edge) is added between the sub-pathways. Ultimately, we removed the pathway steps (purple nodes). This abstraction reveals the high-level sequences between the direct components of a pathway of interest.

In this way, the general succession of biochemical reactions composing the pathway ‘Signaling by EGFR’ is directly visible on the abstracted graph, represented by plain green arrows.

Similarly to this abstraction presented in Fig. 7, different types of abstractions on BioPAX graphs can be developed depending on the analysis. For example, when doing shortest paths analysis on BioPAX data, one would like to abstract the relations of entities to their references (bp3:entityReference properties), which add nodes in the graph.

Overall, the abstraction strategy allows to keep working on BioPAX data when performing specific analysis, while getting rid of a part of its complexity. We thus retain all the advantages of working with a semantic web format (interoperability and standardization), while being able to reason on it and apply classical graph exploration algorithms. One possible future work would be to develop contextual abstraction of BioPAX graphs that would be adapted to specific types of analysis.

## 7 Conclusion

Overall, the BioPAX format answers the initial needs. It provides a controlled vocabulary and a data structure allowing to standardize pathway information. Also, BioPAX improves interoperability between pathway databases, thanks to the ability to map entities to external resources. This capability differentiates BioPAX as an RDF-based format from other systems biology standards such as SBML.

Although BioPAX has been adopted by individual resource providers and aggregation services such as PathwayCommons (Rodchenkov et al. [25]), its use remains very limited. The intrinsic complexity of the format is a hindrance to its wider adoption. BioPAX is designed to be machine-readable, and is thus not directly accessible for researchers without appropriate tools. Moreover, querying BioPAX files needs programming skills (SPARQL query language). Individual data providers could also set up standalone conversion of their data to BioPAX. At present this conversion is only carried out on a regular basis by Reactome. Another area for improvement could be to have more transparent conversion processes from the original data to BioPAX. Currently, there is no way of checking how the original pathway data have been converted in BioPAX in the exports we retrieved from PathwayCommons. In addition, the variability with which BioPAX is used by databases is an obstacle to aggregation procedures of pathway data. There is therefore a need to improve the validation and quality control of BioPAX files. Also, there is an important need of tools allowing to reason on BioPAX graphs, which would be able handle the intrinsic complexity of the format. This is not the case today, as BioPAX files must be often converted into another data format for downstream analyses. To resolve the complexity of BioPAX data, the development of abstraction methods would facilitate the use of BioPAX knowledge graphs by biologists.

## CRediT authorship contribution statement

**Cécile Beust:** Writing – review & editing, Writing – original draft, Formal analysis. **Emmanuelle Becker:** Writing – review & editing, Writing – original draft, Supervision, Conceptualization. **Nathalie Théret:** Writing – review & editing, Writing – original draft, Supervision, Conceptualization. **Olivier Dameron:** Writing – review & editing, Writing – original draft, Supervision, Project administration, Conceptualization.

## Declaration of Competing Interest

The authors have no conflict of interest to declare.

## Data Availability

The codes used in this review, including SPARQL queries, are available as Jupyter notebooks at: https://github.com/CecileBeust/BioPAXReview2024Codes.git.

## Appendix A Supplementary material

MMC Supplementary Materials of the Review Article 'BioPAX in 2024: Where we are and where we are heading' by C. Beust, E. Becker, N. Théret and O. Dameron. MMC
