# Supplementary material for: BioPAX in 2024: Where we are and where we are heading
Source: Comput Struct Biotechnol J. 2024 Nov 4;23:3999–4010. doi: 10.1016/j.csbj.2024.10.045 (PMC11585474; doi:10.1016/j.csbj.2024.10.045)
Supplement: MMC — Supplementary Materials of the Review Article 'BioPAX in 2024: Where we are and where we are heading' by C. Beust, E. Becker, N. Théret and O. Dameron. [file mmc1.pdf]

# Supplementary Materials: BioPAX in 2024: Where we are and where we are heading

Cécile Beust[1], Emmanuelle Becker[1], Nathalie Théret[1,2], and Olivier Dameron[1]

[1]Univ Rennes, Inria, CNRS, IRISA - UMR 6074, F-35000 Rennes, France
[2]Univ Rennes, Inserm, EHESP, Irset, UMR S1085, Rennes, France

Supplementary Figures:

Supplementary Figure S-1: Complete diagram of the BioPAX ontology
Supplementary Figure S-2: RDF triple representation of the pathway 'Formation of RNA Pol II elongation complex' (`R-HSA-112382`) from Reactome (version 90, September 2024)
Supplementary Figure S-3: SPARQL query to extract the pathway 'Formation of the RNA Pol II elongation complex' (`R-HSA-112382`) from Reactome.
Supplementary Figure S-4: Comparison of the contents of BioPAX exports of the nine pathway databases available on PathwayCommons
Supplementary Figure S-5: Mapping process to UniProtKB in the BioPAX export of PANTHER Pathway
Supplementary Figure S-6: Mappings of BioPAX instances to UniProt in the nine pathway databases available on PathwayCommons
Supplementary Figure S-7: Mappings of BioPAX instances to ChEBI in the nine pathway databases available on PathwayCommons
Supplementary Figure S-8: Mindmap/overview of the tools allowing to work with BioPAX data
Supplementary Figure S-9: Simplifcation of the BioPAX graph of the 'Signaling by EGFR' pathway (`R-HSA-177929`) from Reactome (version 90, September 2024)

Supplementary Tables:

Supplementary Table S-1: Comparison table of pathway databases
Supplementary Table S-2: Tools allowing to work with BioPAX files

Supplementary Text:

Supplementary Text S-1: Methods section

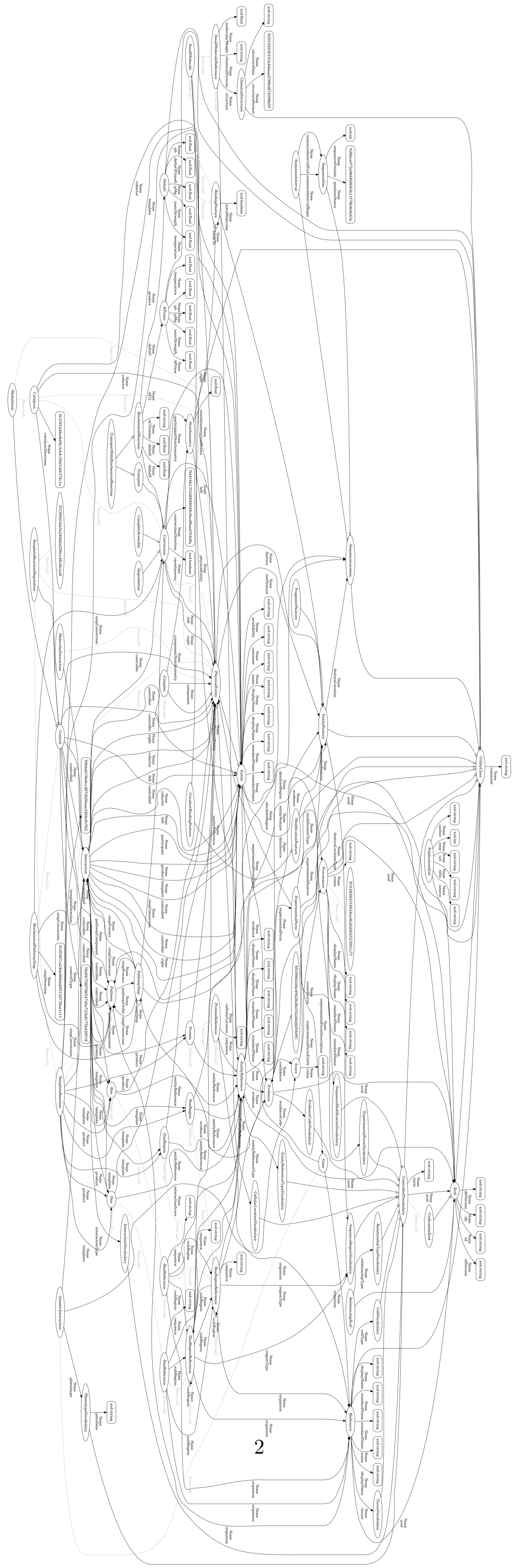

**Supplementary Figure S-1: Complete diagram of the BioPAX ontology**

```
PREFIX rdf: <http://www.w3.org/1999/02/22-rdf-syntax-ns#>
PREFIX rdfs: <http://www.w3.org/2000/01/rdf-schema#>
PREFIX bp3:<http://www.biopax.org/release/biopax-level3.owl#>
PREFIX reactome:<http://www.reactome.org/biopax/77/48887#>

reactome:Pathway1891 rdf:type bp3:Pathway .
reactome:Pathway1891 bp3:displayName "Formation of RNA Pol II elongation complex" .
reactome:Pathway1891 bp3:pathwayOrder reactome:PathwayStep10617 .
reactome:Pathway1891 bp3:pathwayOrder reactome:PathwayStep10618 .
reactome:Pathway1891 bp3:pathwayComponent reactome:BiochemicalReaction8726 .
reactome:Pathway1891 bp3:pathwayComponent reactome:BiochemicalReaction8727 .

reactome:PathwayStep10615 bp3:nextStep reactome:PathwayStep10617 .
reactome:PathwayStep10617 bp3:nextStep reactome:PathwayStep10618 .
reactome:PathwayStep10618 bp3:nextStep reactome:PathwayStep10620 .
reactome:PathwayStep10617 rdf:type bp3:PathwayStep .

reactome:PathwayStep10618 rdf:type bp3:PathwayStep .
reactome:PathwayStep10617 bp3:stepProcess reactome:Catalysis3256 .
reactome:PathwayStep10617 bp3:stepProcess reactome:BiochemicalReaction8726 .

reactome:Catalysis3256 rdf:type bp3:Catalysis .
reactome:Catalysis3256 bp3:controlled reactome:BiochemicalReaction8726 .
reactome:Catalysis3256 bp3:controller reactome:Complex5463 .
reactome:Complex5463 bp3:displayName "P-TEFb complex" .
reactome:Catalysis3256 bp3:controlType "ACTIVATION" .
reactome:Complex5463 bp3:component reactome:Protein15514 .
reactome:Protein15514 bp3:displayName "CDK9" .
reactome:Complex5463 bp3:component reactome:Protein15524 .
reactome:Protein15524 bp3:displayName "CCNT1,CCNT2,CCNK" .

reactome:BiochemicalReaction8726 rdf:type bp3:BiochemicalReaction .
reactome:BiochemicalReaction8726 bp3:displayName "Hyperphosphorylation (Ser2) of RNA Pol II CTD by P-TEFb complex" .
reactome:BiochemicalReaction8726 bp3:left reactome:Complex5463 .
reactome:BiochemicalReaction8726 bp3:right reactome:Complex10863 .
reactome:Complex10863 bp3:displayName "Early elongation complex with hyperphosphorylated Pol II CTD" .

reactome:BiochemicalReaction8727 rdf:type bp3:BiochemicalReaction .
reactome:BiochemicalReaction8727 bp3:displayName "Recruitment of elongation factors to form elongation complex" .
reactome:BiochemicalReaction8727 bp3:left reactome:Complex10863 .
reactome:BiochemicalReaction8727 bp3:right reactome:Complex10864 .
reactome:Complex10864 bp3:displayName "Elongation complex" .
```

**Supplementary Figure S-2: RDF triple representation of the pathway 'Formation of RNA Pol II elongation complex' (R-HSA-112382) from Reactome (version 90, September 2024).**

```
SELECT *
WHERE {
 # Selection of pathway
 VALUES ?pathwayStartName { "Formation of RNA Pol II elongation complex" }
 ?pathwayStart rdf:type bp3:Pathway .
 ?pathwayStart bp3:displayName ?pathwayStartName .

 # Extrat direct pathway components
 ?pathwayStart bp3:pathwayComponent ?pathwayCompo .

 # Extract direct pathway steps
 ?pathwayStart bp3:pathwayOrder ?pathwaySteps .
 ?pathwaySteps rdf:type bp3:PathwayStep .

 # Sequence of pathway steps (via bp3:nextStep property)
 ?previousStep bp3:nextStep ?pathwaySteps .
 ?pathwaySteps bp3:nextStep ?nextStep .

 # Description of step processes
 ?pathwaySteps bp3:stepProcess ?pathwayStepsProcess .
 ?pathwayStepsProcess rdf:type ?pathwayStepsProcessType .

 # Step processes of type BiochemicalReaction
 OPTIONAL {
  ?pathwayStepsProcess bp3:displayName ?pathwayStepsProcessName .
  ?pathwayStepsProcess bp3:left ?left .
  ?pathwayStepsProcess bp3:right ?right .
  OPTIONAL {
   ?left bp3:displayName ?leftName .
   ?right bp3:displayName ?rightName .
   ?left bp3:component ?leftComponent .
   ?leftComponent bp3:displayName ?leftComponentName .
  }
 }

 # Step processes of type Catalysis
 OPTIONAL {
  ?pathwayStepsProcess bp3:controlType ?controlType .
  ?pathwayStepsProcess bp3:controller ?controller .
  ?controller bp3:displayName ?controllerName .
  ?pathwayStepsProcess bp3:controlled ?controlled .
  ?controlled bp3:displayName ?controlledName .
 }
}
```

**Supplementary Figure S-3: SPARQL query to extract the pathway 'Formation of RNA pol II elongation complex' (R-HSA-112382) from the BioPAX export of Reactome.** The BioPAX export of Reactome (version 90, September 2024) is loaded int a SPARQL endpoint and queried in order to retrieve the RDF triples describing the pathway of interest.

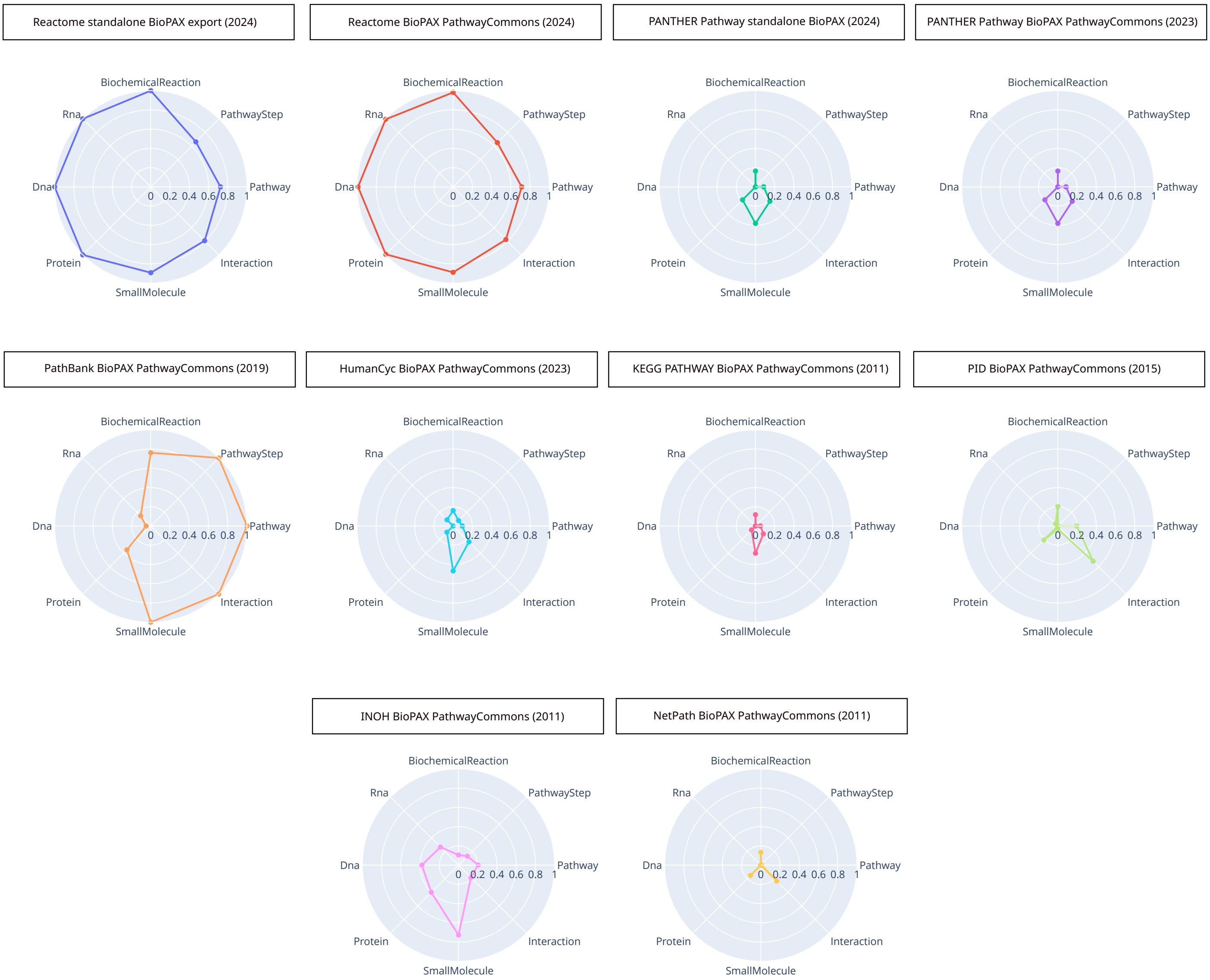


**Supplementary Figure S-4: Comparison of the contents of BioPAX exports of the nine biological pathway databases available on PathwayCommons (Rodchenkov et al., 2019).** The BioPAX exports of nine pathway databases are extracted from PathwayCommons: Reactome, PANTHER Pathway, PathBank, HumanCyc, KEGG Pathway, PID, INOH, NetPath). The standalone BioPAX exports of Reactome (version 90, September 2024) and PANTHER Pathway have been added. For each database, the number of instances of each of the following BioPAX class is represented on the radar plots: `BiochemicalReaction`, `PathwayStep`, `Pathway`, `Interaction`, `SmallMolecule`, `Protein`, `Dna`, `Rna`. The values are calculated as percentages of the maximum value of each category.

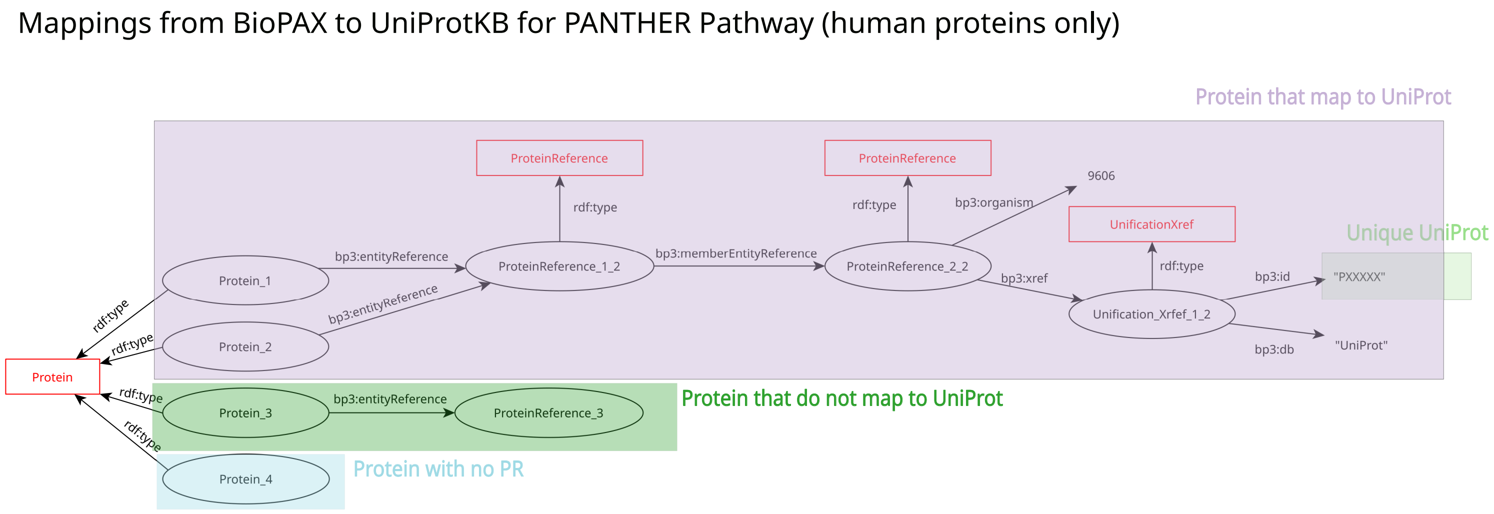


**Supplementary Figure S-5: Mapping process to UniProtKB in the BioPAX export of PANTHER Pathway.** The mappings to UniProtKB are here filtered to get human-only proteins.

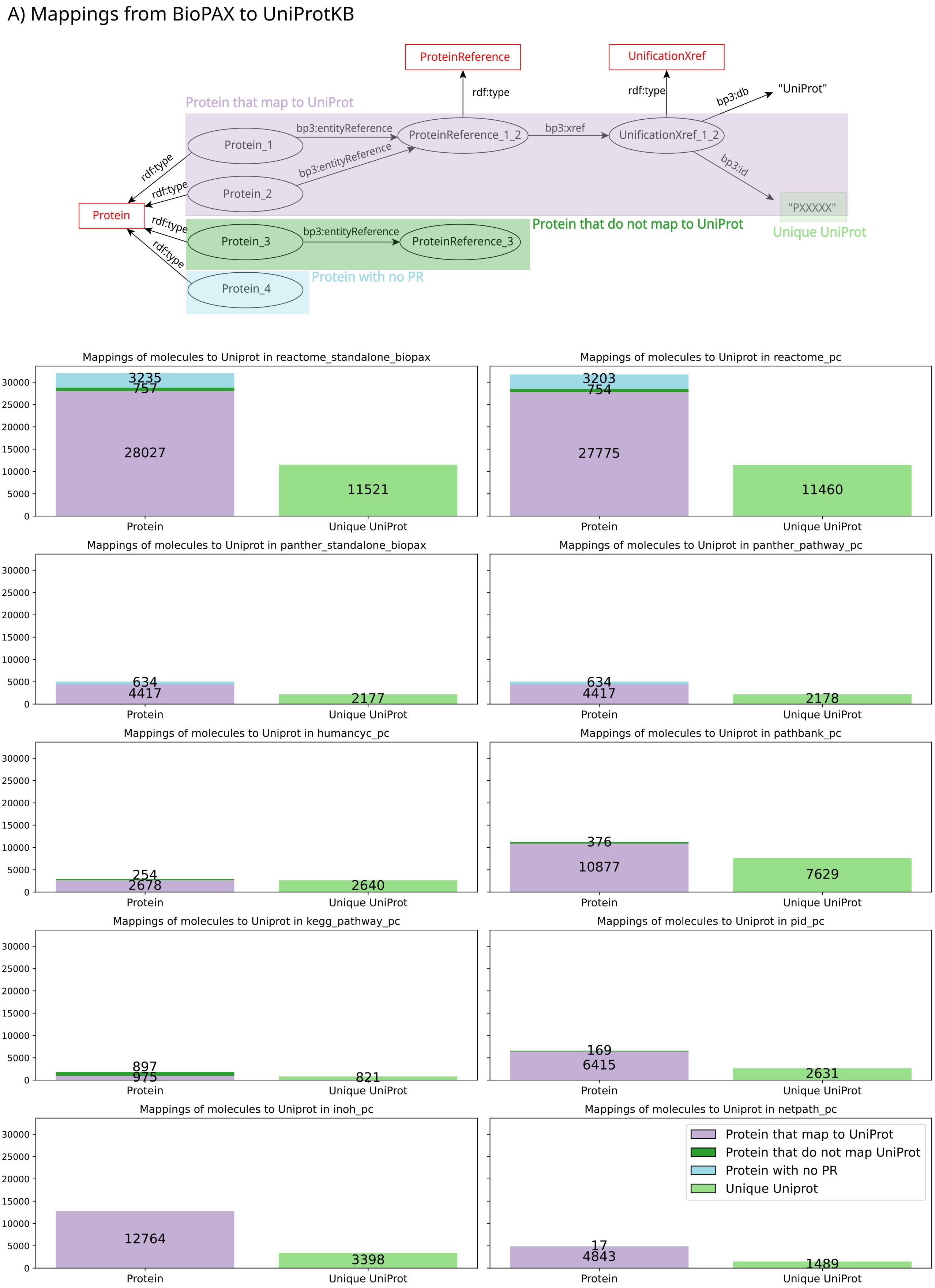


**Supplementary Figure S-6: Mappings of BioPAX instances to UniProt (The UniProt Consortium, 2023) in the BioPAX exports of the nine pathway databases available on PathwayCommons (Rodchenkov et al., 2019)** (Reactome BioPAX version 90, September 2024), Reactome from PathwayCommons, PANTHER Pathway standalone BioPAX export, PANTHER Pathway from PathwayCommons, HumanCyc from PathwayCommons, PathBank from Pathway-Commons, KEGG Pathway from PathwayCommons, PID from PathwayCommons, INOH from PathwayCommons, NetPath from PathwayCommons. The top panel represents the mapping of BioPAX proteins to UniProtKB. Each instance of `Protein` (P) points to `ProteinReference` (PR) that can be linked to a UniProtKB identifier. On the bottom panel, the number of mappings from the BioPAX instances of P to UniProtKB is detailed as well as the number of the unique UniProtKB identifiers that the database points to. The number of instances of P that lack association with PR is also reported, as well as the number of P whose PR do not point to a UniProtKB identifier.

B) Mappings from BioPAX to ChEBI

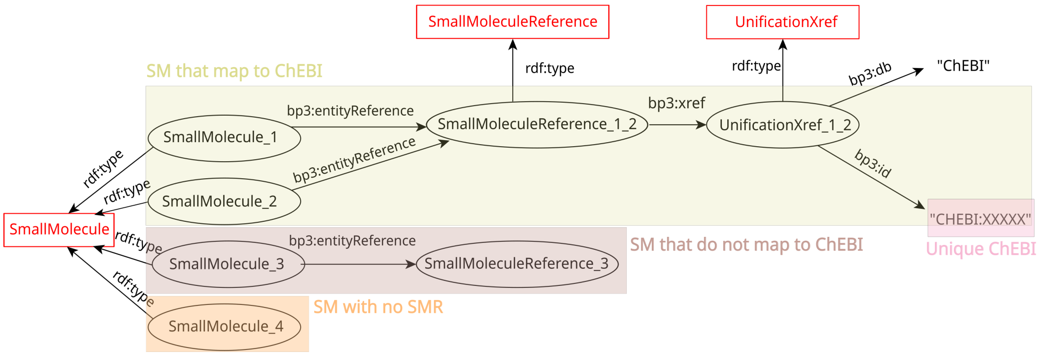


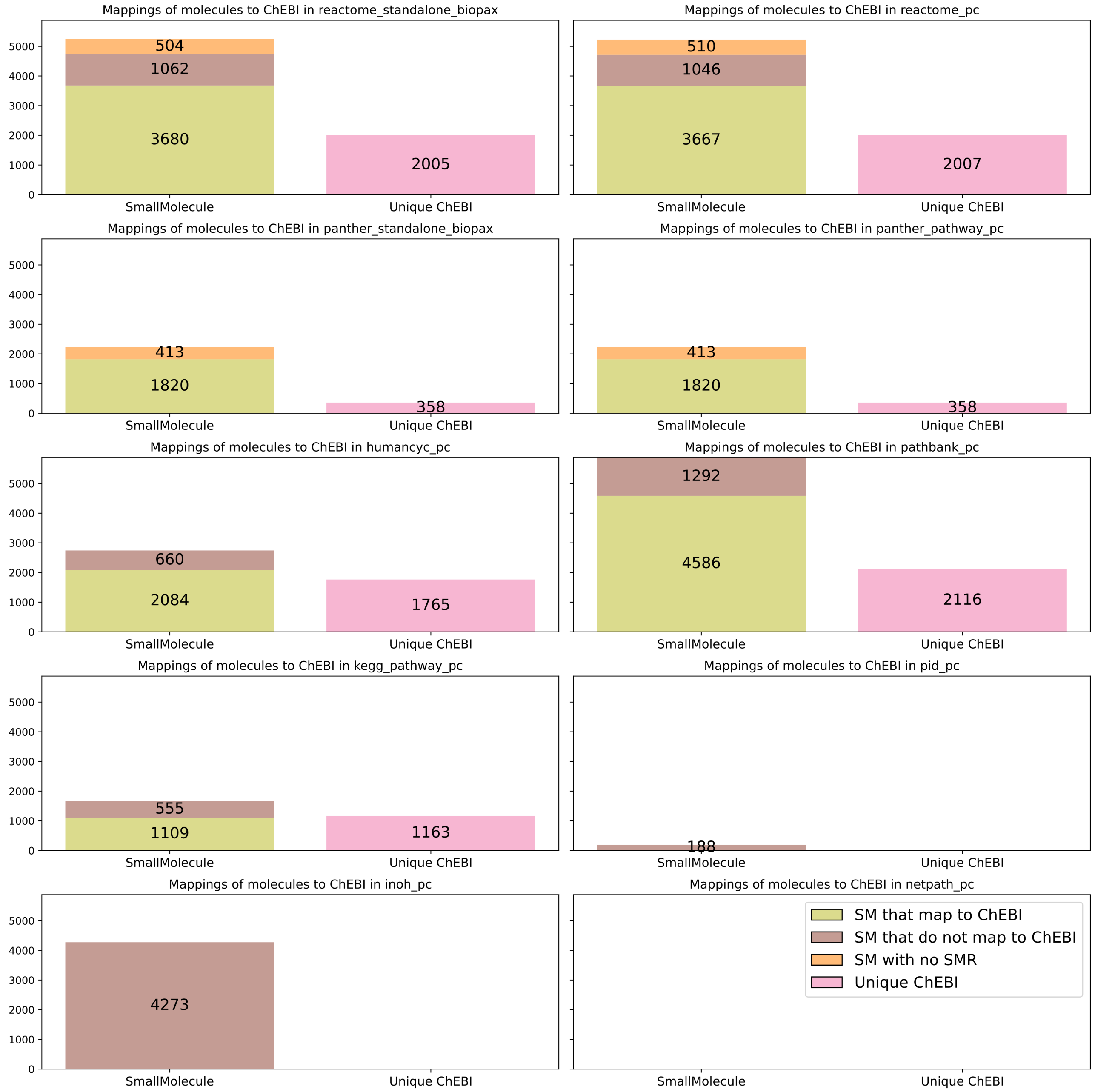


**Supplementary Figure S-7: Mappings of BioPAX instances to ChEBI (Hastings et al., 2016) in the BioPAX exports of the nine pathway databases available on PathwayCommons** (Rodchenkov et al., 2019) (Reactome BioPAX export version 90, September 2024, Reactome from PathwayCommons, PANTHER Pathway standalone BioPAX export, PANTHER Pathway from PathwayCommons, HumanCyc from PathwayCommons, PathBank from PathwayCommons, KEGG Pathway from PathwayCommons, PID from PathwayCommons, INOH from PathwayCommons, NetPath from PathwayCommons. The top panel represents the mapping of BioPAX small molecules to ChEBI. Each instance of `SmallMolecule` (SM) points to `SmallMoleculeReference` (SMR) that can be linked to a ChEBI identifier. On the bottom panel, the number of mappings from the BioPAX instances of SM to ChEBI is detailed as well as the number of the unique ChEBI identifiers that the database points to. The number of instances of SM that lack association with SMR is also reported, as well as the number of SM whose SMR do not point to a ChEBI identifier.

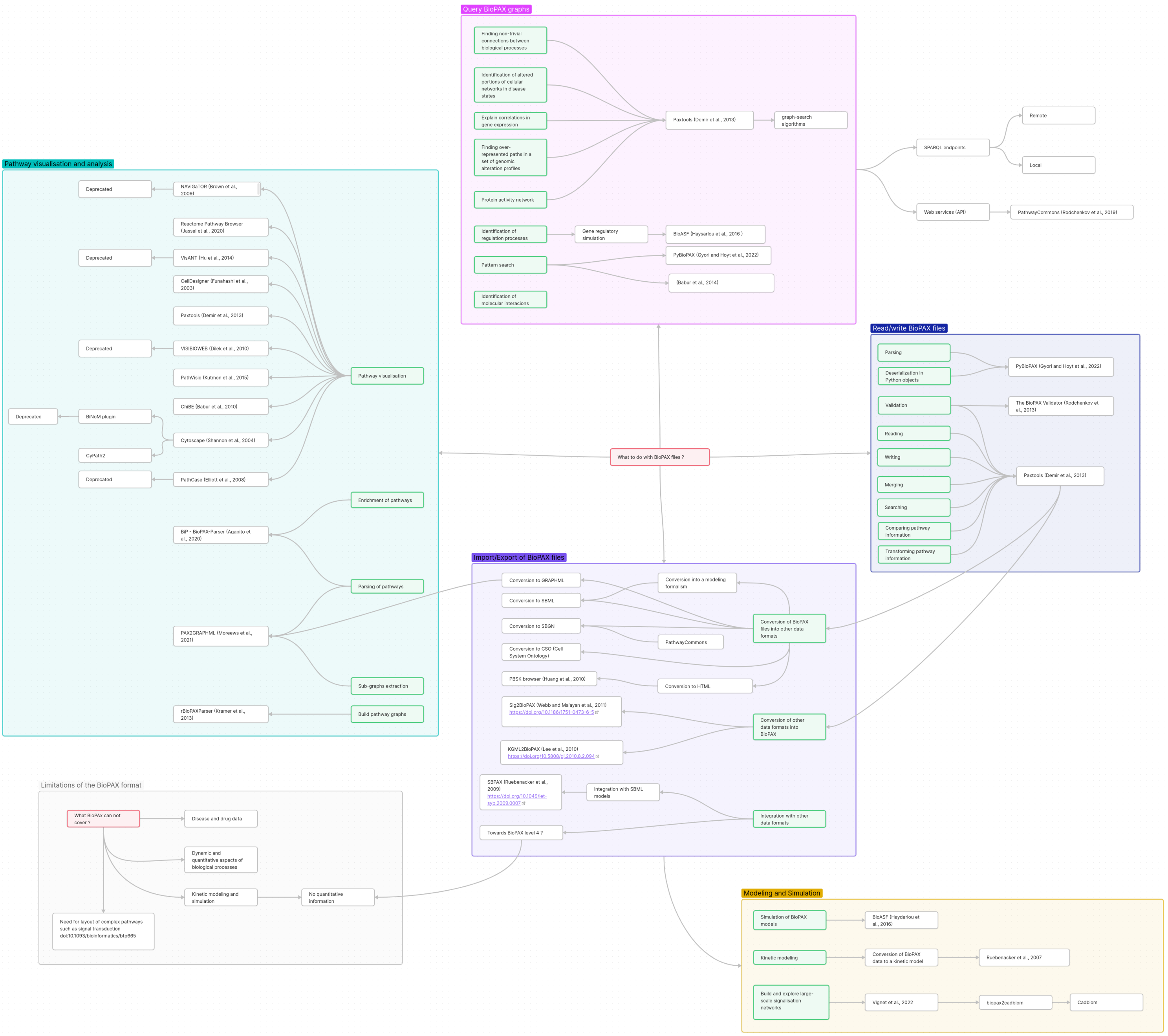


**Supplementary Figure S-8: Mindmap of tools to work with BioPAX files and possible applications**

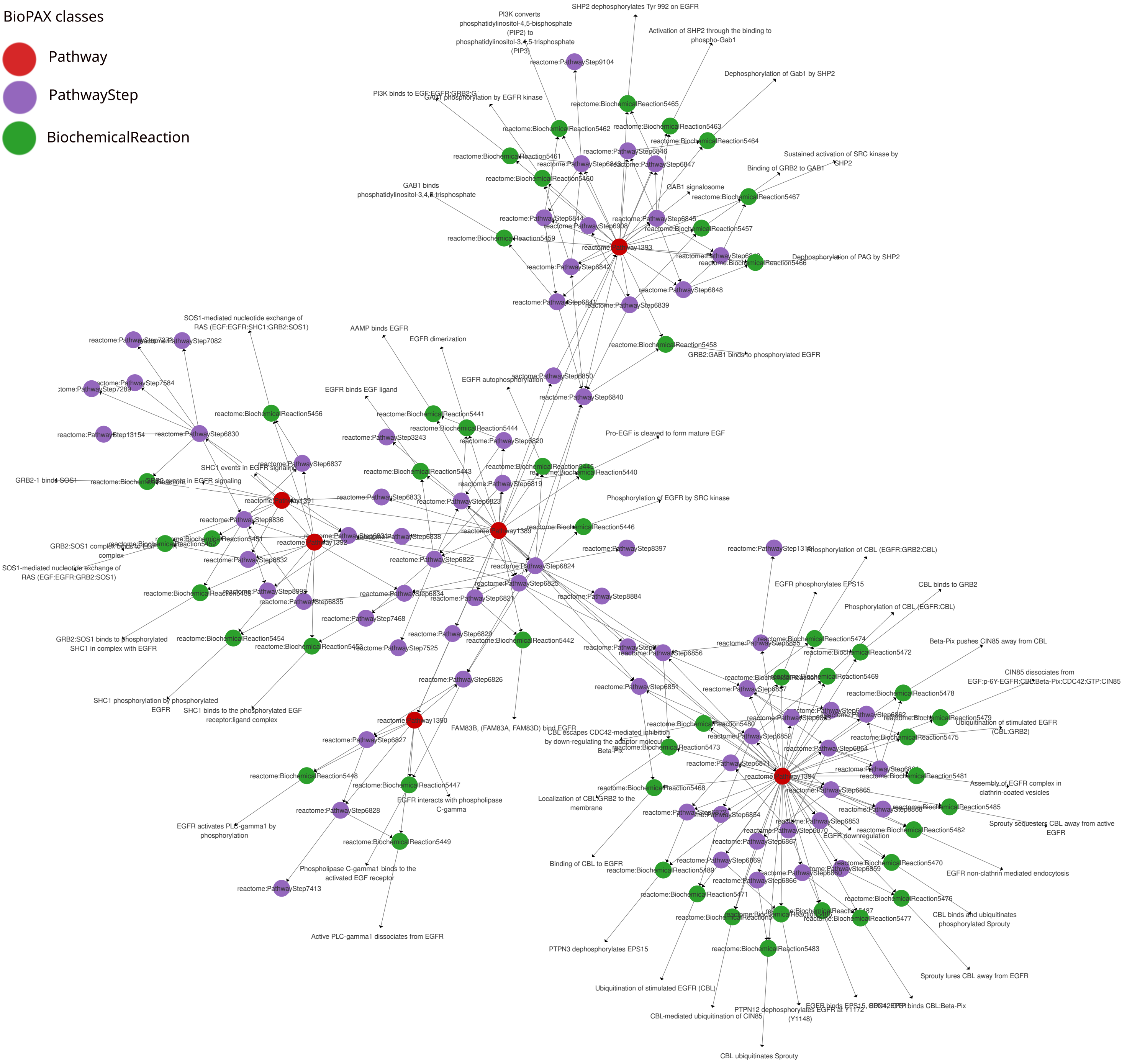


**Supplementary Figure S-9: Simplification of the BioPAX graph of the 'Signaling by EGFR' pathway (R-HSA-177929) from Reactome ('version 90, September 2024).** The BioPAX graph of the pathway was simplified in order to only keep the instances of the `bp3:Pathway`, `bp3:PathwayStep` and `bp3:BiochemicalReaction` classes, represented respectively by red, purple and green nodes.

| Database | **Reactome** | **PANTHER Pathway** | **PathBank** | **HumanCyc** | **KEGG Pathway** | **INOH** | **NetPath** | **PID** |
|---|---|---|---|---|---|---|---|---|
| Website | `https://reactome.org/` | `https://www.pantherdb.org` | `https://pathbank.org/` | `https://humancyc.org/` | `https://www.genome.jp/kegg/pathway.html` | `http://www.inoh.org/` | `http://www.netpath.org/` | `http://pid.nci.nih.gov` |
| Creation | 2002 | 2010 | 2020 | 2005 | 2000 | 2011 | 2010 | 2009 |
| Reference | (Milacic et al., 2024) | (Thomas et al., 2022) | (Wishart et al., 2024) | (Romero et al., 2005) | (Kanehisa and Goto, 2000) | (Yamamoto et al., 2011) | (Kandasamy et al., 2010) | (Schaefer et al., 2009) |
| Scope | Metabolic, signaling and regulatory pathways | Metabolic and regulatory pathways | Metabolite pathway information | Metabolic pathways (human metabolic map) | Metabolic, regulatory, signaling, disease and drug pathways | Signal transduction pathways | Signal transduction pathways | Signaling and regulatory pathways |
| Maintained | Yes | Yes | Yes | Yes | Yes | No | No | No |
| Number of organisms | 15 | 144 | 10 | 1 | 10123 | 5 | 1 | 1 |
| Number of pathways | 2647 | 177 | 605359 | 411 | 1215228 maps | 102 | 10 | 77 |
| Exports | BioPAX, SBML | BioPAX, SBML, SBGN | BioPAX, SBML, PWML, SBGN | BioPAX | KGML, BioPAX, SBML | INOH XML, BioPAX | PSI-MI, SBML, BioPAX | XML, BioPAX |
| PC (v14) File | 2024 | 2023 | 2019 | 2023 | 2011 | 2011 | 2011 | 2015 |

**Supplementary Table S-1: Comparison table of the pathway databases available on PathwayCommons (PC), version 14 (2024)**. Statistics made on July 2024 on the current versions of the databases (when the information were available). The websites of INOH, NetPath and PID are not maintained.

| Tool | Publication | Goal | Input | Output/Exports | Technical specifications | Citations number |
|---|---|---|---|---|---|---|
| Cytoscape | (Shannon et al., 2003) | Network visualization software | SIF, GML, XGMML, SBML, BioPAX, GraphML, Delimited text, Excel Workbook (.xls, .xlsx), Cytoscape.js JSON, Cytoscape CX | CX JSON / CX2 JSON, Cytoscape.js JSON, GraphML, PSI-MI, XGMML, SIF + possibility to save to NDEx | Desktop application, Java implementation | 35522 |
| ChIBE | (Babur et al., 2010, 2014) | Multi-view display, navigation and manipulation of BioPAX models + PathwayCommons queries | PathwayCommons database + local BioPAX files | Static image (in SVG, JPEG, BMP, or PNG file format), GraphML, SIF | Java implementation, Uses Paxtools, Uses SBGN-PD to represent pathway views | 45 |
| PBSK browser | (Huang et al., 2010) | Translate systems biology formats into HTML files | BioPAX, SBML, PSI-MI, KGML | HTML | Web service | |
| KGML2BioPAX | (Lee et al., 2010) | Translation of KGML files to BioPAX | KGML | BioPAX | Java-based conversion modules | 6 |
| CellDesigner | (Funahashi et al., 2008; Mi et al., 2011) | Visualization, modeling and simulation of gene-regulatory and biochemical networks | SBML, SBGN | BioPAX (with BioPAX plugin) | Graphical pathway-editing tool | 629 for CellDesigner + 19 for the BioPAX plugin |
| Sig2BioPAX | (Webb and Ma'ayan, 2011) | Conversion of flat files to BioPAX level 3 | Tabular data describing molecular biochemical reactions | BioPAX level3 | Command-line Java tool | 6 |
| Paxtools | (Demir et al., 2013) | Complete BioPAX API | BioPAX files, Web services | BioPAX, OWL, SBGN-ML, SIF, GSEA | Java implementation | 57 |

| | | | | | | |
|---|---|---|---|---|---|---|
| BioPAX Validator | (Rodchenkov et al., 2013) | Syntactic validation of BioPAX files | BioPAX file | | Java library | 10 |
| rBioPAXParser | (Kramer et al., 2013) | Comprehensive set of functions for parsing, viewing and modifying BioPAX pathway data within R | BioPAX file | BioPAX | R package | 20 |
| PathVisio | (Kutmon et al., 2015) | Pathway editor, visualization and analysis software | Default: GPML Plugins: MIMML (MIM plugin), SBGNML (SBGN plugin), SBML (PathSBML), BioPAX (BioPAX plugin), gene list (MAPPBuilder) | Default: GPML, PNG, PDF, SVG, TIFF, Eu.Gene, datanode list Plugins: MIMML (MIM plugin), SBGNML (SBGN plugin), SBML (PathSBML), HTML (HTMLexporter), BioPAX (BioPAX plugin) | Desktop application, Java implementation | 355 |
| BioASF | (Haydarlou et al., 2016) | Simulation of BioPAX models | BioPAX model + initial physical entity concentrations | Multi-Agent System | Generic simulation framework | 16 |
| PaxtoolsR | (Luna et al., 2016) | Access pathway data in BioPAX in PathwayCommons | BioPAX (local file or from PathwayCommons), SIF, XML | R XML objects | R Package | 25 |
| BIP - BioPAX-Parser | (Agapito et al., 2020) | Parsing of pathways + Pathway Enrichment Analysis (PEA) | BioPAX data from multiple pathway databases (can fetch from PathwayCommons) + Input list of biological entities for PEA | | Graphical software tool | 7 |

| | | | | | | |
|---|---|---|---|---|---|---|
| PAX2GRAPHML | (Moreews et al., 2021) | Generation of graphs of regulated reactions | BioPAX file | Regulated reaction graphs (GraphML) | Python package | 1 |
| PyBioPAX | (Gyori and Hoyt, 2022) | Process and manipulate BioPAX models | BioPAX files + Pathway-Commons client | Deserialization of the BioPAX model into Python classes | Python package | 2 |

**Supplementary Table S-2: Tools allowing to work with BioPAX files**. BioPAX = Biological Pathway Exchange, CX = Cystoscape Exchange , GML = Graph Markup Language, GSEA = Gene Set Enrichment Analysis, HTML = Hypertext Markup Language, KGML = KEGG Markup Language, MIMML = Molecular Interaction Map Markup Language , SBGN = Systems Biology Graphical Notation, SBML = Systems Biology Markup Language, SIF = Simple Interaction Format, PSI-MI = Proteomics Standard Initiative-Molecular Interaction, XGMML = extensible graph markup and modelling language

## Supplementary Text S-1: Methods section

The Jupyter notebooks containing the codes to download BioPAX files, execute the SPARQL queries and build the figures are available on GitHub: `https://github.com/CecileBeust/BioPAXReview2024Codes`

The BioPAX exports of pathway databases were downloaded, either directly from the databases (for Reactome and PANTHER Pathway when we analyze their standalone BioPAX exports) or from the PathwayCommons aggregation service (Rodchenkov et al., 2019). The standalone exports of Reactome and PANTHER Pathway were downloaded on September, 2024. The versions used for analysis are the ones of the standalone exports available at that time (version 90 for Reactome and version file from 2023 for PANTHER Pathway). The BioPAX exports downloaded from PathwayCommons are the ones from the version 14 of the database (July, 2024). BioPAX exports from PathwayCommons are only focused on human data. Concerning the standalone exports, Reactome provides species-specific BioPAX exports, we worked with the human one. For PANTHER Pathway, we downloaded the all-species BioPAX export and then filtered it to only keep human-only protein and remove orthologs.

BioPAX exports of pathway databases were successively loaded into a SPARQL endpoint, with the BioPAX level 3 ontology. The endpoints were created using the Apache Jena Fuseki software (`https://jena.apache.org/documentation/fuseki2/`). 16Go of RAM were allowed to the endpoint to load and query the files. SPARQL queries were executed for each BioPAX file through the SPARQL endpoint and results were obtained in a CSV format (cf GitHub).

The SPARQL queries used to count the number of BioPAX instances (Figure 3) and count the number of mappings for UniProtKB and ChEBI (Figures 4 and 5) are detailed on the associated Jupyter notebooks available on GitHub.

The Cytoscape software (Shannon et al., 2003) was used for to visualize the BioPAX graphs of Figures 6 and 7.

# References


Igor Rodchenkov, Ozgun Babur, Augustin Luna, Bulent Arman Aksoy, Jeffrey V Wong, Dylan Fong, Max Franz, Metin Can Siper, Manfred Cheung, Michael Wrana, Harsh Mistry, Logan Mosier, Jonah Dlin, Qizhi Wen, Caitlin O'Callaghan, Wanxin Li, Geoffrey Elder, Peter T Smith, Christian Dallago, Ethan Cerami, Benjamin Gross, Ugur Dogrusoz, Emek Demir, Gary D Bader, and Chris Sander. Pathway Commons 2019 Update: integration, analysis and exploration of pathway data. *Nucleic Acids Research*, page gkz946, October 2019. ISSN 0305-1048, 1362-4962. doi: 10.1093/nar/gkz946. URL `https://academic.oup.com/nar/advance-article/doi/10.1093/nar/gkz946/5606621`.

The UniProt Consortium. UniProt: the Universal Protein Knowledgebase in 2023. *Nucleic Acids Research*, 51(D1): D523–D531, January 2023. ISSN 0305-1048. doi: 10.1093/nar/gkac1052. URL `https://doi.org/10.1093/nar/gkac1052`.

Janna Hastings, Gareth Owen, Adriano Dekker, Marcus Ennis, Namrata Kale, Venkatesh Muthukrishnan, Steve Turner, Neil Swainston, Pedro Mendes, and Christoph Steinbeck. ChEBI in 2016: Improved services and an expanding collection of metabolites. *Nucleic acids research*, 44(D1):D1214–9, January 2016. ISSN 1362-4962. doi: 10.1093/nar/gkv1031. URL `https://europepmc.org/articles/PMC4702775`.

Marija Milacic, Deidre Beavers, Patrick Conley, Chuqiao Gong, Marc Gillespie, Johannes Griss, Robin Haw, Bijay Jassal, Lisa Matthews, Bruce May, Robert Petryszak, Eliot Ragueneau, Karen Rothfels, Cristoffer Sevilla, Veronica Shamovsky, Ralf Stephan, Krishna Tiwari, Thawfeek Varusai, Joel Weiser, Adam Wright, Guanming Wu, Lincoln Stein, Henning Hermjakob, and Peter D'Eustachio. The Reactome Pathway Knowledgebase 2024. *Nucleic Acids Research*, 52(D1):D672–D678, January 2024. ISSN 1362-4962. doi: 10.1093/nar/gkad1025.

Paul D. Thomas, Dustin Ebert, Anushya Muruganujan, Tremayne Mushayahama, Laurent-Philippe Albou, and Huaiyu Mi. PANTHER: Making genome-scale phylogenetics accessible to all. *Protein Science*, 31(1):8–22, 2022. ISSN 1469-896X. doi: 10.1002/pro.4218. URL `https://onlinelibrary.wiley.com/doi/abs/10.1002/pro.4218`. _eprint: https://onlinelibrary.wiley.com/doi/pdf/10.1002/pro.4218.

David S Wishart, Ray Kruger, Aadhavya Sivakumaran, Karxena Harford, Selena Sanford, Rahil Doshi, Nitya Khetarpal, Omolola Fatokun, Daphnee Doucet, Ashley Zubkowski, Hayley Jackson, Gina Sykes, Miguel Ramirez-Gaona, Ana Marcu, Carin Li, Kristen Yee, Christiana Garros, Dorsa Yahya Rayat, Jeanne Coleongco, Tharuni Nandyala, Vasuk Gautam, and Eponine Oler. PathBank 2.0—the pathway database for model organism metabolomics. *Nucleic Acids Research*, 52(D1):D654–D662, January 2024. ISSN 0305-1048, 1362-4962. doi: 10.1093/nar/gkad1041. URL `https://academic.oup.com/nar/article/52/D1/D654/7420099`.

Pedro Romero, Jonathan Wagg, Michelle L. Green, Dale Kaiser, Markus Krummenacker, and Peter D. Karp. Computational prediction of human metabolic pathways from the complete human genome. *Genome Biology*, 6(1):R2, 2005. ISSN 1474-760X. doi: 10.1186/gb-2004-6-1-r2.

M. Kanehisa and S. Goto. KEGG: kyoto encyclopedia of genes and genomes. *Nucleic Acids Research*, 28(1):27–30, January 2000. ISSN 0305-1048. doi: 10.1093/nar/28.1.27.

Satoko Yamamoto, Noriko Sakai, Hiromi Nakamura, Hiroshi Fukagawa, Ken Fukuda, and Toshihisa Takagi. INOH: ontology-based highly structured database of signal transduction pathways. *Database: The Journal of Biological Databases and Curation*, 2011:bar052, 2011. ISSN 1758-0463. doi: 10.1093/database/bar052.

Kumaran Kandasamy, S Sujatha Mohan, Rajesh Raju, Shivakumar Keerthikumar, Ghantasala S Sameer Kumar, Abhilash K Venugopal, Deepthi Telikicherla, J Daniel Navarro, Suresh Mathivanan, Christian Pecquet, Sashi Kanth Gollapudi, Sudhir Gopal Tattikota, Shyam Mohan, Hariprasad Padhukasahasram, Yashwanth Subbannayya, Renu

Goel, Harrys KC Jacob, Jun Zhong, Raja Sekhar, Vishalakshi Nanjappa, Lavanya Balakrishnan, Roopashree Subbaiah, YL Ramachandra, B Abdul Rahiman, TS Keshava Prasad, Jian-Xin Lin, Jon CD Houtman, Stephen Desiderio, Jean-Christophe Renauld, Stefan N Constantinescu, Osamu Ohara, Toshio Hirano, Masato Kubo, Sujay Singh, Purvesh Khatri, Sorin Draghici, Gary D Bader, Chris Sander, Warren J Leonard, and Akhilesh Pandey. NetPath: a public resource of curated signal transduction pathways. *Genome Biology*, 11(1):R3, 2010. ISSN 1465-6906. doi: 10.1186/gb-2010-11-1-r3. URL https://www.ncbi.nlm.nih.gov/pmc/articles/PMC2847715/.

Carl F. Schaefer, Kira Anthony, Shiva Krupa, Jeffrey Buchoff, Matthew Day, Timo Hannay, and Kenneth H. Buetow. PID: the Pathway Interaction Database. *Nucleic Acids Research*, 37(Database issue):D674–679, January 2009. ISSN 1362-4962. doi: 10.1093/nar/gkn653.

Paul Shannon, Andrew Markiel, Owen Ozier, Nitin S Baliga, Jonathan T Wang, Daniel Ramage, Nada Amin, Benno Schwikowski, and Trey Ideker. Cytoscape : A Software Environment for Integrated Models of Biomolecular Interaction Networks. *Genome Research*, 1(Karp 2001):2498–2504, 2003. doi: 10.1101/gr.1239303.metabolite.

Ozgun Babur, Ugur Dogrusoz, Emek Demir, and Chris Sander. ChiBE: interactive visualization and manipulation of BioPAX pathway models. *Bioinformatics*, 26(3):429–431, February 2010. ISSN 1367-4811, 1367-4803. doi: 10.1093/bioinformatics/btp665. URL https://academic.oup.com/bioinformatics/article/26/3/429/214307.

Özgün Babur, Ugur Dogrusoz, Merve Çakır, Bülent Arman Aksoy, Nikolaus Schultz, Chris Sander, and Emek Demir. Integrating biological pathways and genomic profiles with ChiBE 2. *BMC genomics*, 15(1):642, August 2014. ISSN 1471-2164. doi: 10.1186/1471-2164-15-642.

Donglin Huang, Yiling Huang, Christian Klukas, Ralf Hofestadt, and Ming Chen. PBSK browser: Navigate biological pathways of PSI-MI, BioPAX, SBML and KGML formats. In *2010 IEEE International Conference on Bioinformatics and Biomedicine Workshops (BIBMW)*, pages 13–18, HongKong, China, December 2010. IEEE. ISBN 978-1-4244-8303-7. doi: 10.1109/BIBMW.2010.5703766. URL http://ieeexplore.ieee.org/document/5703766/.

Kyung Eun Lee, Myung Ha Jang, Arang Rhie, Chin Ting Thong, Sanduk Yang, and Hyun Seok Park. Java DOM Parsers to Convert KGML into SBML and BioPAX Common Exchange Formats. *Genomics & Informatics*, 8 (2):94–96, 2010. URL https://genominfo.org/journal/view.php?doi=10.5808/gi.2010.8.2.094. Publisher: Korea Genome Organization.

Akira Funahashi, Yukiko Matsuoka, Akiya Jouraku, Mineo Morohashi, Norihiro Kikuchi, and Hiroaki Kitano. CellDesigner 3.5: A Versatile Modeling Tool for Biochemical Networks. *Proceedings of the IEEE*, 96(8):1254–1265, August 2008. ISSN 1558-2256. doi: 10.1109/JPROC.2008.925458. URL https://ieeexplore.ieee.org/abstract/document/4567412?casa_token=GP5bVuKP-QoAAAAA:aVMgSjVFZsGNhx5r9zGQO7JTtPFRG_HkzoAmk7WBN9xP0d-SnPWc1Flg7QwploDp591aKblp2WoFVg. Conference Name: Proceedings of the IEEE.

Huaiyu Mi, Anushya Muruganujan, Emek Demir, Yukiko Matsuoka, Akira Funahashi, Hiroaki Kitano, and Paul D. Thomas. BioPAX support in CellDesigner. *Bioinformatics (Oxford, England)*, 27(24):3437–3438, December 2011. ISSN 1367-4811. doi: 10.1093/bioinformatics/btr586.

Ryan L. Webb and Avi Ma'ayan. Sig2BioPAX: Java tool for converting flat files to BioPAX Level 3 format. *Source Code for Biology and Medicine*, 6(1):5, March 2011. ISSN 1751-0473. doi: 10.1186/1751-0473-6-5. URL https://doi.org/10.1186/1751-0473-6-5.

Emek Demir, Özgün Babur, Igor Rodchenkov, Bülent Arman Aksoy, Ken I. Fukuda, Benjamin Gross, Onur Selçuk Sümer, Gary D. Bader, and Chris Sander. Using Biological Pathway Data with Paxtools. *PLoS Computational Biology*, 9(9):e1003194, September 2013. ISSN 1553-7358. doi: 10.1371/journal.pcbi.1003194. URL https://dx.plos.org/10.1371/journal.pcbi.1003194.

Igor Rodchenkov, Emek Demir, Chris Sander, and Gary D. Bader. The BioPAX Validator. *Bioinformatics*, 29(20): 2659–2660, October 2013. ISSN 1367-4803. doi: 10.1093/bioinformatics/btt452. URL `https://www.ncbi.nlm.nih.gov/pmc/articles/PMC3789551/`.

Frank Kramer, Michaela Bayerlová, Florian Klemm, Annalen Bleckmann, and Tim Beißbarth. rBiopaxParser—an R package to parse, modify and visualize BioPAX data. *Bioinformatics*, 29(4):520–522, February 2013. ISSN 1367-4811, 1367-4803. doi: 10.1093/bioinformatics/bts710. URL `https://academic.oup.com/bioinformatics/article/29/4/520/197940`.

Martina Kutmon, Martijn P. van Iersel, Anwesha Bohler, Thomas Kelder, Nuno Nunes, Alexander R. Pico, and Chris T. Evelo. PathVisio 3: An Extendable Pathway Analysis Toolbox. *PLOS Computational Biology*, 11(2): e1004085, February 2015. ISSN 1553-7358. doi: 10.1371/journal.pcbi.1004085. URL `https://journals.plos.org/ploscompbiol/article?id=10.1371/journal.pcbi.1004085`. Publisher: Public Library of Science.

Reza Haydarlou, Annika Jacobsen, Nicola Bonzanni, K. Anton Feenstra, Sanne Abeln, and Jaap Heringa. BioASF: a framework for automatically generating executable pathway models specified in BioPAX. *Bioinformatics*, 32(12): i60–i69, June 2016. ISSN 1367-4811, 1367-4803. doi: 10.1093/bioinformatics/btw250. URL `https://academic.oup.com/bioinformatics/article/32/12/i60/2240600`.

Augustin Luna, Özgün Babur, Bülent Arman Aksoy, Emek Demir, and Chris Sander. PaxtoolsR: pathway analysis in R using Pathway Commons. *Bioinformatics*, 32(8):1262, April 2016. doi: 10.1093/bioinformatics/btv733. URL `https://www.ncbi.nlm.nih.gov/pmc/articles/PMC4824129/`. Publisher: Oxford University Press.

Giuseppe Agapito, Chiara Pastrello, Pietro Hiram Guzzi, Igor Jurisica, and Mario Cannataro. BioPAX-Parser: parsing and enrichment analysis of BioPAX pathways. *Bioinformatics*, 36(15):4377–4378, August 2020. ISSN 1367-4803. doi: 10.1093/bioinformatics/btaa529. URL `https://doi.org/10.1093/bioinformatics/btaa529`.

François Moreews, Hugo Simon, Anne Siegel, Florence Gondret, and Emmanuelle Becker. PAX2GRAPHML: a python library for large-scale regulation network analysis using BioPAX. *Bioinformatics*, 37(24):4889–4891, December 2021. ISSN 1367-4803, 1367-4811. doi: 10.1093/bioinformatics/btab441. URL `https://academic.oup.com/bioinformatics/article/37/24/4889/6299382`.

Benjamin M. Gyori and Charles Tapley Hoyt. PyBioPAX: biological pathway exchange in Python. *Journal of Open Source Software*, 7(71):4136, March 2022. ISSN 2475-9066. doi: 10.21105/joss.04136. URL `https://joss.theoj.org/papers/10.21105/joss.04136`.
